# A Comparison of Entropic Diversity and Variance in the Study of Population Structure

**DOI:** 10.3390/e25030492

**Published:** 2023-03-13

**Authors:** Eric F. Karlin

**Affiliations:** School of Theoretical & Applied Science, Ramapo College, Mahwah, NJ 07430, USA; ekarlin@ramapo.edu

**Keywords:** AMOVA, allele metric diversity, diversity, almost unbiased estimators, F_ST_, G_ST_, Jost’s D, D_EST_, expected heterozygosity, population structure, q = 1, q = 2, Shannon Index, Shannon informational diversity translation analysis, variance

## Abstract

AMOVA is a widely used approach that focuses on variance within and among strata to study the hierarchical genetic structure of populations. The recently developed Shannon Informational Diversity Translation Analysis (SIDTA) instead tackles exploration of hierarchical genetic structure using entropic allelic diversity. A mix of artificial and natural population data sets (including allopolyploids) is used to compare the performance of SIDTA (a ‘q = 1’ diversity measure) vs. AMOVA (a ‘q = 2’ measure) under different conditions. An additive allelic differentiation index based on entropic allelic diversity measuring the mean difference among populations (Ω_AP_) was developed to facilitate the comparison of SIDTA with AMOVA. These analyses show that the genetic population structure seen by AMOVA is notably different in many ways from that provided by SIDTA, and the extent of this difference is greatly affected by the stability of the markers employed. Negative among group values are lacking with SIDTA but occur with AMOVA, especially with allopolyploids. To provide more focus on measuring allelic differentiation among populations, additional measures were also tested including Bray–Curtis Genetic Differentiation (BCGD) and several expected heterozygosity-based indices (e.g., G_ST_, G″_ST_, Jost’s D, and D_EST_). Corrections, such as almost unbiased estimators, that were designed to work with heterozygosity-based fixation indices (e.g., F_ST_, G_ST_) are problematic when applied to differentiation indices (eg., D_EST_, G″_ST_, G′_ST_H).

## 1. Introduction

The genetic structure of populations has been studied by many approaches, and these have been considered to fall into two classes: fixation measures (e.g., F_ST_, G_ST_) and allelic differentiation measures (e.g., Jost’s D, D_EST_, entropy differentiation) [1]. The two classes have been shown to focus on different aspects of population structure, with the former primarily reflecting the relative degree of fixation present, and the latter focusing on the relative extent of differentiation [1]. In contrast to fixation measures, the development and application of allelic differentiation measures in populations has been recent, occurring primarily within the past 15 years [2,3,4,5,6,7,8,9,10]. Not surprisingly, there have been several studies comparing the strengths and weaknesses of these two classes [11,12,13,14,15,16]. However, prior to the advent of allelic differentiation measures, fixation measures were, and still are, often misconstrued as being differentiation measures [1], and I am among the many who have had this misinterpretation at some point in my career.

Analysis of Molecular Variance (AMOVA) [17] is a widely used method which employs variance to study the hierarchical genetic structure of populations. It yields F statistics including F_ST_, which is a widely used, and oldest, measure of population structure [1]. As F_ST_ may also be based on heterozygosity, I use F_STv_ to refer to variance-based F_ST_ and F_STh_ to refer to heterozygosity-based F_ST_. Both AMOVA and heterozygosity-based indices are q = 2 measures. Recently an entropic differentiation measure using allelic diversity based on Shannon informational diversity translation analysis (SIDTA, which is a q = 1 measure) to explore the hierarchical genetic structure of populations has been articulated [2,3,4,5,6,7,8,9,10].

As AMOVA (variance based) and SIDTA (based on entropic q = 1 allelic diversity) are used to study the genetic hierarchical structure of populations, it would be useful to compare their respective outcomes, particularly given the confusion about just what F_STv_ measures. SIDTA expresses allelic diversity (D) within a stratum as the mean effective number of alleles (EFNA) within a stratum (e.g., effective number of alleles within a population) at a given marker and allelic diversity between strata as the effective number of subgroups within a group (e.g., effective number of populations within a region) at a given marker [3,4,8]. The product of these values yields the grand total of EFNA (Equation (1)), and SIDTA-based allelic diversity is thus multiplicative (Equation (1)). The hierarchical population structure based upon SIDTA, adapted from [8], is shown in Table 1. D′ is a differentiation measure which converts D values into [0, 1] scaled proportions of the theoretical maximum diversity possible with a given data set [8]. When sample size is balanced across all populations, D′_AP_ is calculated by Equation (2) (where k = number of populations).

D_T_ = (D_WI_·D_AI_·D_AP_·D_AR_) (1)

D′_AP_ = (1 − (1/D_AP_))/(1 − (1/k)) (2)

In contrast, AMOVA yields additive results, using mean estimated variance per haplotype to refer to variance both within a stratum and between strata. This difference complicates a direct comparison of the two approaches. One way around this problem is by translating the multiplicative ‘among strata’ diversity components of D (e.g., effective number of subgroups within a group) into an equivalent, but additive, effective number of alleles among subgroups within a group (e.g., mean effective number of alleles among populations within a region). This approach is described and implemented in two recent studies [18,19]. These additive ‘between strata’ diversity analogues are referred to as allelic-metric diversity (AMD, denoted as Δ) to distinguish them from the typical multiplicative between strata diversity components (denoted as D). The equations for the calculation of Δ are shown in Table 2. The relationship between the multiplicative D among group indices and the additive Δ among group is shown in Equation (10). This slight tweaking of SIDTA allows for a more in-depth exploration of the hierarchical structure of populations as well as for a more direct comparison with AMOVA, as well as with other measures having additive results (e.g., heterozygosity-based fixation indices such as F_STh_ and G_ST_). 

**Table 2:** Hierarchical population structure based on AMD (Δ). (EFNA: effective number of alleles).

Allelic Diversity within Strata (as Effective Number of Alleles)	Allelic Diversity between Strata [Additive] (as Effective Number of Alleles)	
D_T_ = grand total EFNA	Δ_AR_ = mean EFNA among regions	
	Δ_AR_ = (D_T_ − D_WR_)	(6)
D_WR_ = mean EFNA within regions	Δ_AP_ = mean EFNA among pops. within regions	
	Δ_AP_ = (D_WR_ − D_WP_)	(7)
D_WP_ = mean EFNA within pops.	Δ_AI_ = mean EFNA among inds. within pops.	
	Δ_AI_ = (D_WP_ − D_WI_)	(8)
D_WI_ = mean EFNA within inds.	Δ_TAP_ = total EFNA among pops.	
	Δ_TAP_ = Δ_AP_/((k − 1)/k)	(9)

D_T_ = (D_WI_·D_AI_·D_AP_·D_AR_) = (D_WI_ + Δ_AI_ + Δ_AP_ + Δ_AR_) (10)

Another approach to calculate the AMD (Δ) among groups is shown in Equation (11):

Δ_AP_ = ((D_AP_ − 1)·D_WP_) = (D_AP_ −1)·D_AI_·D_WI_ (11)

In this study, I compare the entropic q = 1 level SIDTA approach using AMD, with a variance-based approach (AMOVA) in the exploration of the genetic structure in populations, with a focus on all of the components of genetic structure, not just the difference among populations. The comparison is across different levels of marker variability and across ploidy levels. Given its emphasis in many studies, several other indices estimating difference among populations are included for comparison with both Δ_AP_ and F_STv_ (based on variance). Seven of these other indices are based on expected heterozygosity, which uses a q = 2 approach. They include: F_STh_, G_ST_, G′_ST_N (Nei’s standardized G_ST_), G′_ST_H (Hedrick’s standardized G_ST_), G″_ST_ (Hedrick’s standardized G_ST_, further corrected for bias when population number is small), Jost’s D, and D_EST_. Additionally, included is Allele Frequency Difference (AFD) [20,21], an approach based on either allele frequency or relative allele frequency data and hereafter referred to as Bray–Curtis Genetic Difference (BCGD).

Finally, a further goal is to present the information in a way that allows readers who are not statisticians to more easily grasp and understand the outcomes.

## 2. Materials and Methods

### 2.1. Formats Used to Present Difference among Populations 

Three formats are commonly used to present the difference between strata by studies of the genetic hierarchical structure of populations. They are the mean difference among groups (MDAG, [0, 1] scaled): mean difference among populations = MDAP; mean difference among regions = MDAR; the total difference among groups (TDAG, [0, 1] scaled); and the effective number of groups (ENG [1, G] scaled, with G = the number of groups). For differences among populations these formats would be: (1) the mean difference per population expressed as a proportion of the total difference in a given data set (MDAP format: e.g., F_STv_, F_STh_, G_ST_); (2) the overall total (not mean per population) difference among all populations expressed as a proportion of the theoretical maximum difference possible (i.e., [0, 1] scaled indices) for a given data set (TDAP format: e.g., D′_AP_, F′_STv,_ G′_ST_H, Jost’s D. D_EST_, BCGD); (3) the overall total difference among all populations expressed as the effective number of populations (‘ENP’ format: e.g., D_AP_). The TDAP indices are based on the total difference among populations (and not the mean difference among populations) and are thus optimal for differentiation studies. In contrast, the MDAP format provides information directly relating to the hierarchical structure of populations. Two weaknesses of the MDAP format are (1) that it only shows a portion of the total difference among populations, and (2) the commonly used heterozygosity- and variance-based MDAP indices (i.e., F_STv_, F_STh_, G_ST_) are fixation measures, not differentiation measures.

### 2.2. Nomenclature for ‘MD’ Formatted Indices with SIDTA

As noted above, AMOVA has both ‘MDAP’ formatted (e.g., F_STv_) and TDAP-formatted indices (F′_STv_) for among populations. By design SIDTA has ENP formatted (i.e., D_AP_) and TDAP-formatted (i.e., D′_AP_) indices, but lacks an MDAP-formatted index. With AMD (Δ), however, the creation of an additive ‘MDAP’ formatted index is possible. Thus, to facilitate the comparison of SIDTA with the ‘MDAP’ formatted F_STv_ associated with AMOVA, an MD-formatted system of measures (referred to as ‘Ω’) based on Δ values was developed for SIDTA data (Table 3). In contrast to MDAP-formatted indices based on heterozygosity and variance (e.g., F_STv_, F_STh_, and G_ST_), which are fixation measures, Ω_AP_ is a [0–0.5] scaled differentiation measure representing the mean allelic diversity among populations expressed as a proportion of the grand total diversity for a given data set (D_T_) (Equation (12)). The range of values and expected behavior for Ω and the other indices used in this study are presented in Supplemental Table S2.

In addition to Equation (2), when all populations have a balanced number of samples, the TDAP index D′_AP_ can also be calculated based on both Δ data and Ω data (Equations (16) and (17)). Thus, these three equations (Equations (2), (16) and (17)) all yield the same result.

D′_AP_ = Δ_TAP_/D_T_ (16)

D′_AP_ = Ω_AP_/((k − 1)/k) (17)

D′_AP_ can, in turn, be apportioned among populations to yield the MDAP-formatted Ω_AP_. For a data set without a regional stratum, and when all populations (k) have an equal number of samples, Ω_AP_ can be directly derived from D′_AP_ by Equation (18):

Ω_AP_ = D′_AP_·((k − 1)/k) (18)

In addition to the traditional F values, I use F_T_ to refer to the grand total variance. I also use F_WI_ and F_AI_ to refer to the proportion of F_T_ represented by variance within individuals and by variance among individuals within populations, respectively. N_a_ stands for the grand total number of different alleles in a data set (or subset) and is a q = 0 diversity index. N_a-MIN_ is the theoretical minimum (N_a-MIN_ = 1 = D_T-MIN_) for a given data set (or subset) and N_a-MAX_ stands for the theoretical maximum N_a_ possible (N_a-MAX_ = D_T-MAX_). For data sets I–III, each subset has (N_a-MAX_ = 40 = D_T-MAX_)_._ Finally, N′_a_ is the proportion of N_a-MAX_ present in a given data set (or subset). 

### 2.3. Data Sets

#### 2.3.1. Data Set I (DS-I): No Allelic Overlap between Each Pair of Artificial Populations in a Subset and with Ω_AP_ = 0.50 for Each Subset

Nine pairs (subsets) of artificial diploid populations were created, each representing two populations and based on one ‘marker’. Each subset had the following properties: ten samples per population; no allelic overlap between populations, and with equal AMD (Δ), equal heterozygosity, and equal variance within each population. By design, Ω_AP_ was 50% for each subset, and the corresponding TDAP-formatted indices were at the theoretical maximum (i.e., F′_STv_ = 1.0 = D′_AP_). The N′_a_ for the subsets ranged from 10% to 90% of N_a-MAX_ (i.e., subsets had 10%, 20%, …, 90% of N_a-MAX_). Two additional subsets were created, one having N_a-MIN_ and one having N_a-MAX_. This data set is available online in Supplemental File S1.

#### 2.3.2. Data Set II (DS-II): Allelic Overlap between Each Pair of Artificial Populations in a Subset, with Ω_AP_ Varying across Subsets

Ten (10) pairs of artificial populations (subsets) were created based on the same parameters as the subsets in DS-I with the exceptions of (1) allelic overlap occurring between the two populations in each subset, and (2) TDAP indices were not at the theoretical maximum. This data set is available online in Supplemental File S1.

#### 2.3.3. Data Set III (DS-III): Allelic Overlap between the Two Populations in Each Subset Plus an Imbalance in Heterozygosity, Variance, and the Δ between Them

Eleven (11) pairs of artificial populations (subsets) were created based on the same parameters used for the subsets in DS-II except that heterozygosity, variance, and Δ are not balanced between the two populations in each subset. The N′_a_ for the subsets ranged from 0.05–0.95 (i.e., subsets had 5%, 10%, 20%, …, 90%, 95% of N_a-MAX_). All of the changes in N′_a_ were limited to one population (population A) across the subsets having N′_a_ = 0.90–0.50, with the second population (population B) being unchanged across these subsets. The change in N′_a_ for subset N′_a_ = 0.40 resulted from changes in N_a_ made in both populations. For this subset, there was no variance and heterozygosity in the population A (i.e., N_a_ = 1.0). For subsets N′_a_ = 0.3–0.05, change in N′_a_ was limited to population B. This pattern yields an infection point at N′_a_ = 0.5. Change in N_a_ was required in both populations to achieve N′_a_ = 0.95. This data set is available online in Supplemental File S1.

#### 2.3.4. Data Sets Based on Natural Populations

The first three data sets were designed as stress tests to see how well SIDTA and AMOVA performed in extreme conditions and included the heterozygosity-based difference ‘among population’ indices as well as BCGD. To compare the findings of the artificial data sets with that of natural populations, SIDTA and AMOVA were run on microsatellite (SSR) data sets from prior studies on *Sphagnum* (peat moss) gametophytes: haploid data set [22], gametophytically allodiploid data set [23,24], gametophytically allotriploid data set [18], and a ‘semi-natural’ diploid data set. The latter was created by pairing haploid haplotypes (from 22) of one species to make diploid genotypes and then arbitrarily placing the genotypes into two ‘semi-natural’ populations. In one semi-natural population, different haplotypes were paired. This was also followed in part for the second population, which also included some pairing of haplotype copies (to allow for the occurrence of intragametophytic fertilization). For each ploidy level, the SSRs were grouped into highly stable SSRs (STAB subset), moderately variable SSRs (MOD subset), and hypervariable SSRs (HYPE subset), with each subset being analyzed separately. The SSR data sets are available online in Supplemental File S2.

### 2.4. Mathematical Analyses

AMOVA and Shannon informational diversity translation analysis (SIDTA) were carried out on each natural data set using GenAlEx 6.52b1 [25,26,27]. The ENP formatted between strata values (‘D’) were converted to AMD values (both Δ and Ω) by hand, following the method described in [18] and outlined in the Introduction. Data sets I–III were also analyzed by BCGD and several heterozygosity-based indices (all but one implemented and documented by GenAlEx, where they are placed under the G statistics tab). With the exception of F_STh_ and Jost’s D, calculations of the other heterozygosity-based indices are adjusted (estimated) by GenAlEx by applying the corrections for small population size (almost unbiased estimations) [28] and inbreeding [29] in the calculations of H_S_ (mean heterozygosity within populations) and H_T_ (total heterozygosity pooled across populations). The adjusted indices are: G_ST_, G′_ST_N (Nei’s standardized G_ST_), G′_ST_H (Hedrick’s standardized G_ST_), G″_ST_ (Hedrick’s standardized G_ST_, further corrected for bias when population number is small), and D_EST_. In addition to providing almost unbiased estimations for these indices, two other outcomes of this adjustment are that it (1) allows for the possibility of negative values with indices having this adjustment, and, (2) for analyses with just one marker, results in F_STh_ have higher values than those of the corresponding adjusted G_ST_ and also for Jost’s D to have higher values than D_EST_. As they were not an option with the GenAlEx implementation of SIDTA, almost unbiased estimators were not used with the SIDTA-based indices. Jost’s D was calculated manually in Excel using sample frequency data generated by GenAlEx.

An additional index, the Bray–Curtis index of dissimilarity [30] was also included. Originally formulated to assess differences between communities based on species importance values, this index has also been used to measure allelic differentiation between populations, using either an allele frequency format or a proportion format in place of species importance values (albeit with each format requiring different, but equivalent, equations) [20,21]. I will refer to this approach as Bray–Curtis genetic differentiation (BCGD). BCGD may be expressed in both [0, 1] scaled TDAP format (BCGD, Equation (19)) and a [0, 2] scaled (BCGD2, Equation (20)) format. Calculation of BCGD followed an approach noted in [31] and used by [20], which is analogous to the approach based on species importance values presented in [32]. Calculations of BCGD values were performed manually using Excel and applied to the proportions (relative frequency) of alleles generated by GenAlEx. The proportion of an allele in one population is referred to as ‘p_1_’ and its proportion in the second population as ‘p_2_’.

[0, 1] scaled BCGD = 1 − ∑ min (p_1_ or p_2_) = 0.5∙∑|(p_1_ − p_2_)| (19)

[0, 2] scaled BCGD2 = ∑|(p_1_ − p_2_)| (20)

Linear regression comparing selected indices against N′_a_ across the subsets of a data set was calculated using Excel.

## 3. Results

To start off, as implemented by GenAlEx, it was found that (F_STv_ = G′_ST_N) and (F′_STv_ = G″_ST_) across all of the data sets analyzed for this study. This indicates a close relationship between the variance-based AMOVA and heterozygosity-based indices. Accordingly, for the balance of the paper, F_STv_ and F′_STv_ will each also represent the corresponding G statistic. In addition, G′_ST_N is clearly an MDAP-formatted fixation measure.

### 3.1. Analyses of Data Set 1

#### 3.1.1. Among Population Indices

##### MDAP-Formatted Indices

**Figure 1:**
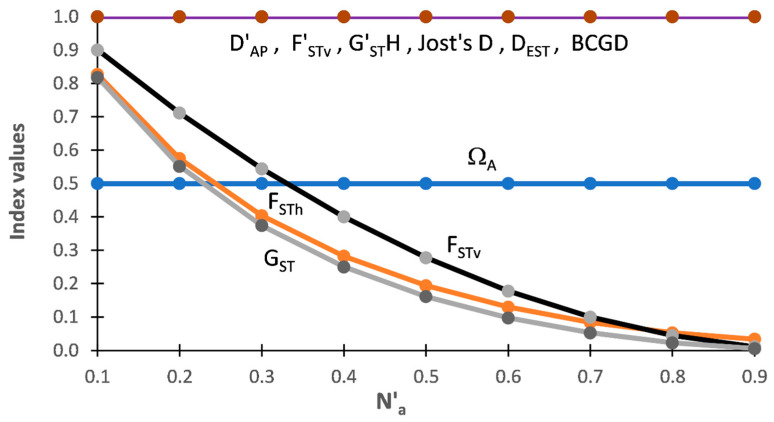
Results of five MDAP and seven TDAP indices based on SIDTA, AMOVA, and heterozygosity measures across a wide range of N′_a_ for the nine pairs of artificial populations (subsets) of Data Set I (DS-I). Values for the MADP indices (Ω_AP_, F_STv_ (=G′_ST_N), F_STh_, G_ST_) measure the mean proportion of total difference between two populations and the values for the TDAP indices (D′_AP_, F′_STv_ (=G″_ST_), G′_ST_H, Jost’s D, D_EST_, BCGD) measure the proportion of the theoretical maximum difference between two populations. The MDAP-formatted Ω_AP_ measures both.

Figure 1 shows the outcome of the analyses for among population indices based on DS-I. One thing that clearly stands out is that Ω_AP_ and F_STv_ are unequivocally measuring different aspects of population structure. Ω_AP_ is constant at 50% of the N′_a_ across the range of allelic diversity present within these subsets while F_STv_ ranges from 1.0 (near N_a-MIN_) to 0.0 (at/near N_a-MAX_). At the minimal allelic diversity (N_a-MIN_), when there is just one allele across both populations, Ω_AP_ = 0 while F_STv_ is undefined (online Supplemental Table S1). The pattern for Ω_AP_ (Figure 1) shows that it is a differentiation measure. Based on DS-I, Ω_AP_ is independent of the level of D_T_ when there is no allelic overlap among populations. In sharp contrast, F_STv_ has an inverse relationship with the level of N′_a_ present in each subset, being highest at minimal levels of N’a and having a sigmodal decrease as N′_a_ increases. At N′_a-MAX_, when every individual in a subset has unique alleles for a given marker, all pairwise comparisons are identical and net variance only occurs within individuals (online Supplemental Table S1). In other words, at N′_a-MAX_ AMOVA is completely blind to the corresponding Δ_AI_ and Δ_AP_ that is present. This pattern indicates that F_STv_ is a fixation measure that tracks the relative degree of fixation in each population pair and that is strongly affected by the level of D_T_. In strong contrast, Ω_AP_ shows that each population has half of the total allelic diversity present in each subset. Although measuring different things, Ω_AP_ ≈ F_STv_ when N′_a_ = 0.03 to 0.035 (Figure 1), indicating that the extent of differentiation (Ω_AP_) and degree of fixation (F_STv_) have comparable values around this level of N′a with DS-I.

##### TDAP-Formatted Indices

By design F′_STv_ and D′_AP_ both = 1.0 across all subsets (Figure 1) and these two TDAP-formatted indices, consequently, fail to show that the two approaches are measuring different aspects of population structure with this data set.

##### Other among Population Indices

Results for the other ‘among population’ indices show that F_STh_ and G_ST_ both follow a pattern across the nine subsets similar to that shown by F_STv_ (=G′_ST_N) (Figure 1). This indicates that, similar to F_STv_, the heterozygosity-based MTAP-formatted indices are also tracking the fixation present among populations. As with D′_AP_ and F′_STv_ (=G″_ST_), the four other TDAP indices (G′_ST_H, Jost’s D, D_EST_, and BCGD) all equal ‘1.0’ across the nine subsets with this data set.

#### 3.1.2. Among Individuals within Populations (Ω_AI_ and F_AI_)

**Figure 2:**
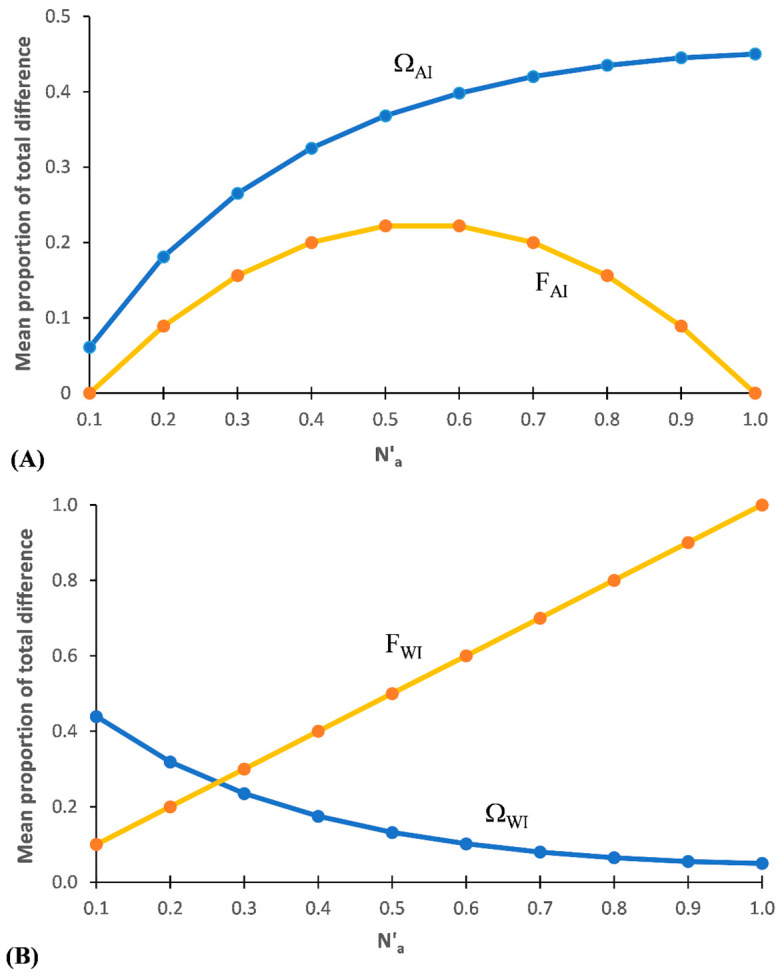
Mean proportion of total allelic diversity and total variance represented based on Data Set I. (**A**) among individuals within populations (Ω_AI_ and F_AI_, respectively), and (**B**) within individuals (Ω_WI_ and F_WI_, respectively) between ten pairs of ‘artificial’ diploid populations across a wide range of N′_a_.

The percentage of total D_T_ and F_T_ represented among individuals nested within populations (Ω_AI_ and F_AI_, respectively) across the maximum range of N_a_ possible with these subsets is shown in Figure 2A. F_AI_ shows a pattern similar to that of an optimum response curve, with F_AI_ = 0.0 at/near N_a min_ and also at/near N_a-MAX_ and peaking at F_AI_ ≈ 0.22 in subsets around N′_a_ ≈ 0.55%. At low values for N′_a_ in a subset, both variance among individuals (F_AI_) and that within individuals (F_WI_) rise (Figure 2B) as N′_a_ increases. However, as N′_a_ continues to increase, a point is reached where a large percentage of the combinations of the different alleles present results in the maximum value for the basal stratum (F_WI_). This saturation redundancy for F_WI_ is one reason why AMOVA is blind to allelic diversity. Further increases in N′_a_ will lead to both increasing levels of saturation redundancy and also to decreasing levels of variance among all individuals (F_IT_) until the only net variance remaining is that within individuals (occurring when D_T_ = N′_a-MAX_).

The pattern for Ω_AI_ indicates that it measures differentiation among individuals, with Ω_AI_ increasing as D_T_ increases, reaching a peak at N′_a_ = 1. Thus, every allele is counted and the problem of saturation redundancy associated with AMOVA is lacking with SIDTA. Ω_AI_ exceeds the corresponding F_AI_ value across the subsets, with the divergence between the two measurements increasing notably at very high levels of D_T_. When Ω_AI_ is maximal with DS-I (at N′_a_ = 1.0) the corresponding value is F_AI_ = 0.0 (Supplemental Table S1).

#### 3.1.3. Within Individuals (Ω_WI_ and F_WI_)

With diploid data, the proportion of total variance found within individuals (‘F_WI_’) increases as N′_a_ increases, with ‘F_WI_’ = 1.0 at N′_a_ = 1.0 (=N_a-MAX_) for these data sets (Figure 2B). This pattern suggests that F_WI_ is tracking the extent of fixation, but in the opposite direction that is measured by F_STv_. In contrast, the proportion of D_T_ found within individuals (Ω_WI_) follows a curvilinear decrease with increasing N′_a_, with Ω_WI_ = 1.0 at the theoretical minimum N′_a_ and decreasing τo Ω_WI_ = 0.05 at the maximum (N′_a_ = 1) with these data sets (Supplemental Table S1).

### 3.2. Analyses of Data Set II

**Figure 3:**
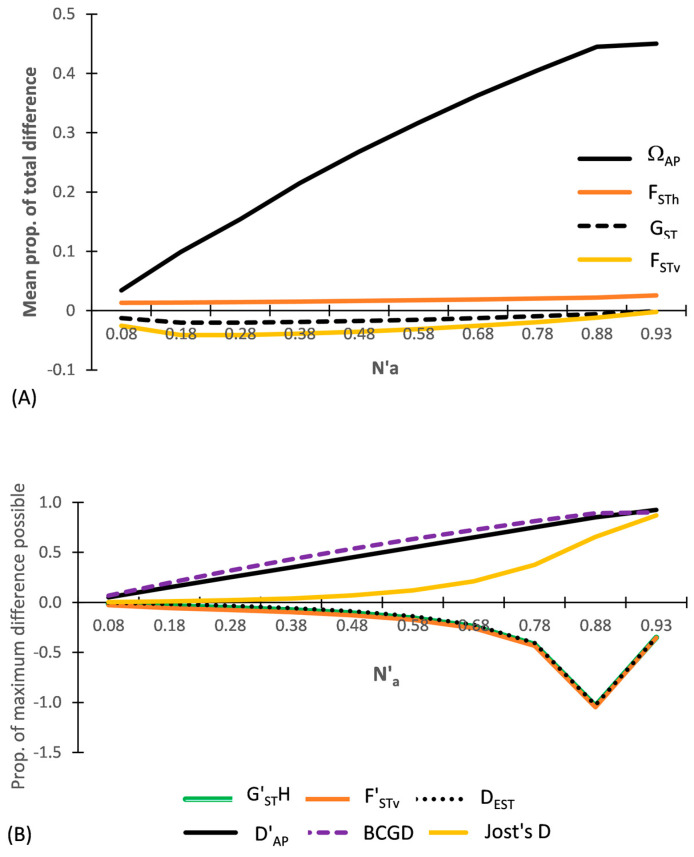
Results of five MDAP and seven TDAP indices based on SIDTA, AMOVA, and heterozygosity measures across a wide range of N′_a_ for the ten pairs of artificial populations (subsets) of Data Set II (DS-II). (**A**) Values for the MADP indices with (F_STv_ = G′_ST_N) and (**B**) values for the TDAP indices (with F′_STv_ = G″_ST_). The MDAP-formatted Ω_AP_ represents both the mean proportion of total difference between two populations and the mean maximum difference possible between two populations. G′_ST_H lies between F’_STv_ and D_EST_ and is barely visible.

The results for DS-II are shown in Figure 3, which focuses only on among population indices. By having the level of heterozygosity and variance balanced between each population pair, the differentiation between each population pair is minimal. Thus F_STh,_ F_STv_, and G_ST_ are minimal (close to ‘0’) across all subsets. A distinct demarcation exists between the three entropic based diversity indices (Ω_AP_, D′_AP_, D_AP_ (not shown) and also by BCGD, which are all positive while, with the exception of F_STh_ and Jost’s D, all of the other heterozygosity and variance-based indices (F_STv,_ G_ST_, G′_ST_H, F′_STv_, and D_EST_) are all negative (Figure 3). The presence of positive and negative values among the various heterozygosity-based indices for the same subset is addressed in Section 4. 

#### 3.2.1. MDAP Indices

Ω_AP_ closely tracks changes in N′_a_, being half of D′_AP_ and positive across all subsets (Figure 3A). In contrast, F_STv_ is negative and very close to ‘0.0’ across all subsets, differing greatly from Ω_AP_. Divergence between these two indices increases notably as N′_a_ approaches N′_a_-_MAX_. The heterozygosity-based MDAP indices closely follow the same pattern as that shown by F_STv_, with all values tracking very close to 0.0 across all subsets. However, the unadjusted F_STh_ is positive across all subsets while the adjusted heterozygosity MDAP indices (G_ST_ and G′_ST_N (=F_STv_)) are slightly negative across all subsets. These patterns clearly show the difference between what the fixation indices (F_STv_ (=G′_ST_N), F_STh_, G_ST_) measure and what is measured by Ω_AP_, which is a differentiation measure.

#### 3.2.2. TDAP Indices

By design, the TDAP indices vary across the subsets with DS-II (Figure 3B). D′_AP_ is twice as large as Ω_AP_ (Figure 3A) and closely tracks changes in allelic diversity as N′_a_ increases across the subsets. In striking contrast to the results of DS-I, D′_AP_ differs notably from all of the TDAP-formatted heterozygosity- and variance-based indices (Figure 3B). Jost’s D stands out from the adjusted heterozygosity-based TDAP indices in being positive across all subsets of DS-II, showing a sigmoidal increase as N′_a_ increases, with a prolonged slow rate of increase across subsets having a low N′_a_ (Figure 3B). Notably, this sigmodal response, which is typical of q = 2-based diversity indices, lies well below the corresponding D′_AP_ and BCGD values for all of the subsets, except near the two end points. It even falls below Ω_AP_ up until N′_a_ = 0.78. F′_STv_ is negative and becomes more negative as N’_a_ increases across the subsets. G′_ST_H, G″_ST_, and D_EST_ diverge from the pattern shown by Jost’s D as N′_a_ increases across the subsets and instead closely track F′_STv_, with (G″_ST_ = F′_STv_). BCGD closely tracks the level of N′_a_ across the nine subsets, yielding values very close to those of D′_AP_. Linear regression shows that of the TDAP indices, BCGD has the tightest fit with N′_a_ (y = x − 0.0025; R^2^ = 1.0; *p* < 0.001), with D′_AP_ falling very close behind (y = 1.0245x + 0.0256; R^2^ = 0.9938; *p* < 0.001).

### 3.3. Analyses of Data Set III

#### 3.3.1. MDAP-Formatted Indices

**Figure 4:**
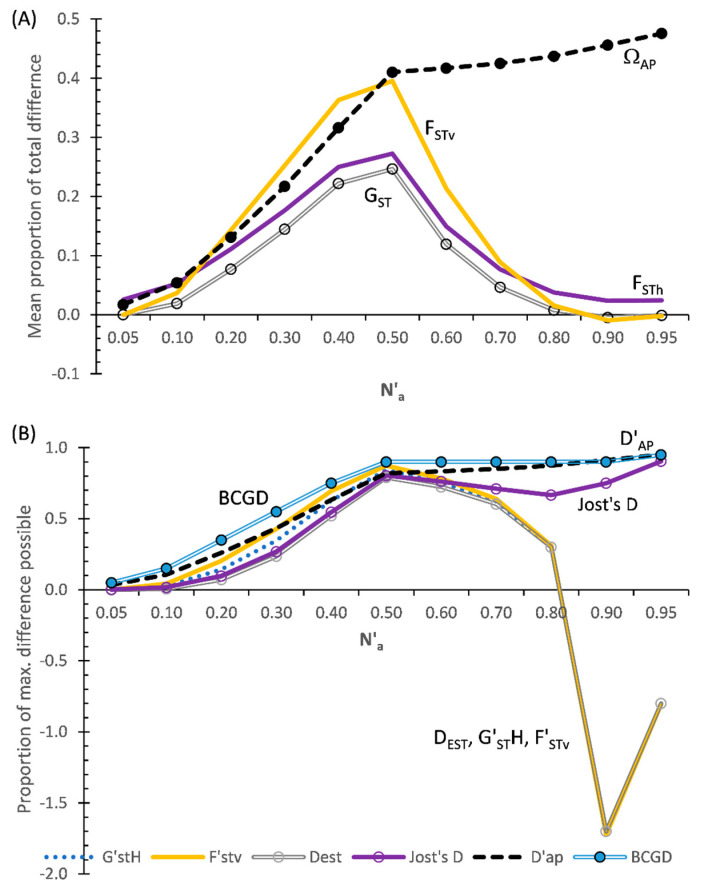
Results of five MDAP and seven TDAP indices based on SIDTA, AMOVA, and heterozygosity across a wide range of N′_a_ across 11 pairs of artificial populations based on Data Set III (DS-III): (**A**) MDAP indices (with G′_ST_N = F_STv_) and (**B**) TDAP indices (with G″_ST_ = F′_STv_). The MDAP-formatted Ω_AP_ represents both the mean proportion of total difference between two populations and the mean maximum difference possible between two populations.

Figure 4A shows the results for the MDAP-based indices based on Data Set III. What stands out in this figure is that although Ω_AP_ increases as N′_a_ increases across all subsets of DS-III, F_STv_ at first increases as N′_a_ increases and it then decreases abruptly above N′_a_ = 0.5. The three MDAP heterozygosity-based indices (F_STh_, G_ST_, and G′_ST_N) follow a similar pattern, or an identical pattern in the case of G′_ST_N, to that of F_STv_. As seen with DS-II (Figure 3A), the adjusted G_ST_ closely tracks F_STh_ across all subsets (Figure 4A). In comparison with the heterozygosity-based values (except for G′_ST_N), F_Stv_ more closely compares to Ω_AP_ between N′_a_ = 0.2–0.5 with this data set than either F_STh_ and G_ST_ do. Both F_STv_ and G_ST_ become slightly negative above N′_a_ = 0.8, while F_STh_ remains positive across all subsets. This pattern conforms with the information in Supplemental Table S2.

#### 3.3.2. TDAP-Formatted Indices

As found with DS-II, D′_AP_ differs notably from all of the TDAP-formatted heterozygosity and variance-based indices with DS-III (Figure 4B). D′_AP_ increases as N′_a_ increases across all subsets. Although comparable to D′_AP_ across all subsets, the TDAP-formatted BCGD flatlines (at BCGD = 0.9) across subsets N′_a_ = 0.5–0.9. In contrast, F′_STv_ and the heterozygosity-based indices all show both increases and decreases across the subsets of DS-III (Figure 4B), with F_STh_ always being positive while F_STv_ and the adjusted heterozygosity-based indices are both positive and negative.

All of the TDAP indices coincide around N′_a_ = 0.5, occurring due to one population having very low diversity (e.g., high homozygosity) at that point, while the second population has a very high allelic diversity (high heterozygosity). F′_STv_ most closely matches D′_AP_ up to N′_a_ = 0.5 and then greatly deviates from that index. After closely tracking Jost’s D up to N′_a_ = 0.5, the adjusted heterozygosity indices (D_EST_ G′_ST_H, and G″_ST_) above that point all greatly diverge from Jost’s D and instead closely track F′_STv_ (Figure 4B).

### 3.4. Analyses of Natural Populations

The patterns of genetic structure associated with the three data sets using ‘artificial’ populations are unique to those data sets. Although differing from the patterns associated with the artificial populations in some respects, analyses of the natural population data sets all show Ω_WI_ decreasing with increases in N’_a_.

#### 3.4.1. Monomorphic Markers

To study the influence of monomorphic markers on SIDTA and AMOVA, analysis of STAB subsets of haploid data was undertaken (Supplemental File S2). One set of ten STAB SSRs (STAB-10) included two monomorphic SSRs. The second set (STAB-8) had the same SSRs excluding the monomorphic SSR-16 and SSR-19. Because AMOVA sums variance across markers, monomorphic markers, for which variance = 0, do not affect F_ST_. Consequently, F_ST_ is identical with both the STAB-8 and STAB-10 sets (F_ST_ = 0.53). In contrast, Ω_AP_ = 0.26 based on the STAB-8 set and Ω_AP_ = 0.21 with the STAB-10 set. This difference results from two things: (1) monomorphic markers have a diversity value of 1.0, and (2) SIDTA averages the diversity across markers. Consequently, the presence of monomorphic markers in a data set has the potential to reduce Ω_AP_. The larger the proportion of monomorphic markers included in a data set the greater the potential reduction. However, the inclusion of monomorphic markers in a data set allows for that level of diversity to be counted in studies on genetic structure. With the exception of the STAB-10 set used here and the data set showing theoretical minimum allelic diversity (where there is just one allele present), all of the data sets used in this study lack monomorphic SSRs to allow for a more direct comparison of SIDTA and AMOVA.

#### 3.4.2. Haploid Data Set

**Figure 5:**
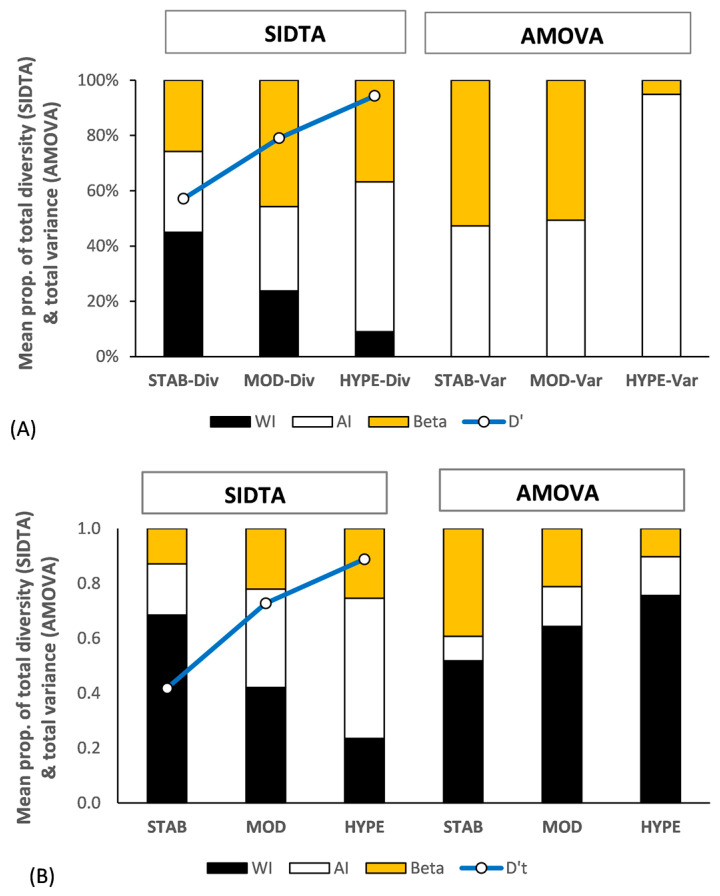
Population genetic structure (black: within individuals; no fill: among individuals within populations; orange: between populations) yielded by SIDTA and AMOVA and based on STAB, MOD, and HYPE SSR subsets for haploid and diploid populations. (**A**) between regional populations of haploid gametophytes of *Sphagnum comosum* and *S. novo-zelandicum* (14 samples each) and (**B**) between regional artificial populations of diploid sporophytes of *S. novo-zelandicum* (6 samples each). The [0, 1] scaled diversity (D′_T_) for the total allele metric diversity detected with each data set is shown by the blue line. Beta refers to both Ω_AP_ and F_STv_.

Figure 5A shows the genetic structure across the three data sets for the haploid gametophytes of *Sphagnum comosum* Müll. Hal. and of *S. novo-zelandicum* Mitt. (14 samples each). The relationship between Ω_AP_ and F_STv_ basically conforms with the pattern seen in Figure 1, with (Ω_AP_ ≪ F_STv_) at lower N′_a_ values, (Ω_AP_ ≈ F_STv_) at moderate N′_a_ values, and (Ω_AP_ ≫ F_STv_) at very high N′_a_ values. The extent of fixation between populations (F_STv_) greatly exceeds the differentiation between populations (Ω_AP_) with the STAB subset, with the reverse being the case with the HYPE set. D′_T_ for the three data sets is 0.49 for the STAB subset, 0.79 for the MOD subset, and 0.94 for the HYPE subset. Both Ω_AP_ and F_STv_ are significant across all three data sets (α = 0.05) with *p* values for Ω_AP_ being more significant with the MOD and HYPE subsets than the corresponding *p* values for F_STv_.

As there is typically no variance within haploid individuals, all variance is among individuals with AMOVA at that ploidy level. In contrast, Ω_WI_ = 1 with haploid data, and this allows for allelic diversity to occur both within and among individuals. This increases the difference between F_STv_ and Ω_AP_ values with haploid data, particularly at lower levels of N′_a_. However, as Ω_WI_ = 1 across the STAB, MOD, and HYPE subsets, this difference is largely offset by the higher levels of N′_a_ associated with the MOD and HYPE subsets (Figure 5A).

#### 3.4.3. Diploid Data Set

Figure 5B shows the genetic structure of two artificial diploid populations of *Sphagnum novo-zelandicum* (six samples each) based on both SIDTA and AMOVA across the three SSRsub sets. With diploid data, both SIDTA and AMOVA have the potential for a within individual stratum. The diploid data follow the same general pattern present in the haploid data, with the exception of the inclusion of the ‘within individual’ stratum with AMOVA. There is an increase in Ω_AP_ and a corresponding decrease in F_STv_ as N′_a_ increases, with Ω_WI_ and F_WI_ following the opposite pattern along that gradient. As with the haploid data, (Ω_AP_ ≪ F_STv_) at lower N′_a_ values, (Ω_AP_ ≈ F_STv_) at moderate N′_a_ values, and (Ω_AP_ > F_STv_) at very high N′_a_ values. The diploid SIDTA data differs from the haploid data in that Ω_WI_ is higher and Ω_AP_ is lower than that for the haploid data across the three data sets. The increase in Ω_AIT_ as N’_a_ increases mostly occurs in Ω_AI_, while the decrease in F_IT_ as N’_a_ increases lies in F_STv_. The diploid AMOVA data shows that the majority of variance within populations occurs within individuals with this data set. Both Ω_ST_ and F_STv_ are significant (α = 0.05) for the STAB and MOD subsets but differ with the HYPE subset, with the *p* value being not significant for Ω_AP_ (*p* = 0.206) and significant with the corresponding *p* value for F_ST_ (*p* = 0.014).

#### 3.4.4. Allopolyploid Data Sets

**Figure 6:**
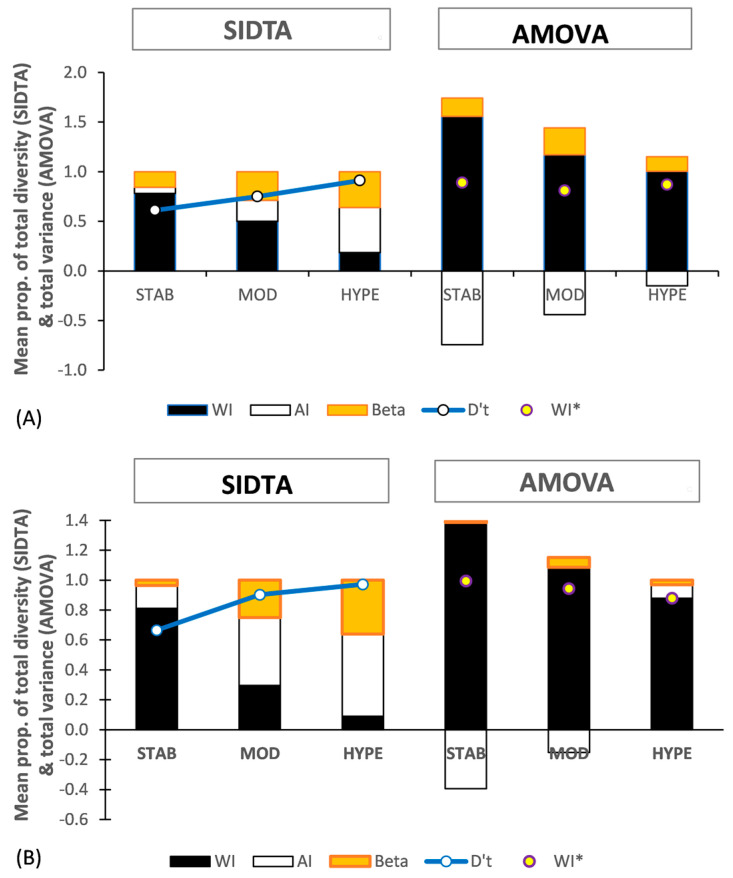
Population genetic structure (black: within individuals; no fill: among individuals within populations; orange: between populations) yielded by SIDTA and AMOVA and based on STAB, MOD, and HYPE SSR sets for allodiploid and allotriploid data. (**A**) regional populations of two gametophytically allodiploid species (South Island, NZ population of *Sphagnum × cristatum* and Hawaiian population of *S.× palustre*) and (**B**) two South Island, NZ populations of the gametophytically allotriploid *Sphagnum × falcatulum*. The [0, 1] scaled diversity (D′) for the total allele metric diversity detected with each data set is shown by the blue line. Beta refers to both Ω_AP_ and F_STv_. WI* refers to F_WI_* which is calculated by ignoring negative F_AI_ values (i.e., treating them as 0.0).

Figure 6 shows the population structure based on SIDTA and AMOVA across three SSR subsets for gametophytic populations of allopolyploid *Sphagnum* species. Figure 6A is based on regional populations of two gametophytically allodiploid *Sphagnum* species: the Hawaiian population of *S. × palustre* L. and the South Island, NZ population of *S. ×cristatum* Hampe, each with 31 samples. Figure 6B is based on two South Island, New Zealand populations of the gamtetophytically allotriploid *Sphagnum × falcatulum* Besch., each with f21 samples. AMOVA yields negative F_AI_ values for both allopolyploid data sets, resulting in complex and puzzling population structures. In strong contrast, lacking negative values, SIDTA shows that both the allopolyploids follow the same general pattern for population structure as seen for SIDTA applied to the non-allopolyploid data sets: with (1) Ω_WI_ decreasing with increasing allelic diversity, and (2) Ω_AP_ increasing along the same gradient (Figure 6).

Based on AMOVA, negative estimated variance among individuals nested within populations occurs and it is included in the calculation of F_STv_, F_AI_, and F_WI_ for both ploidy levels, resulting in negative F_AI_ values. Negativity in F_AI_ decreases as marker variability increases. While negative F_AI_ values occur across all three SSR subsets with the allodiploid data, they are limited to the STAB and MOD SSR subsets with the allotriploid data (Figure 6A,B). The greatly distorted population structure that AMOVA sees in the presence of negative F values is evident in these figures. For instance, the presence of negative values results in F_WI_ > 1.0. With AMOVA, negative F_AI_ values arise when the mean square within individuals exceeds the mean square among individuals nested within populations. Finally, both the allodiploid and allotriploid data sets show F_WI_ being highest with the STAB subset and declining as SSR variability increases, the opposite pattern of that seen in the non-allopolyploid data sets. 

For comparison purposes, F statistics based on negative estimated variance among individuals nested within populations treated as 0.0 were also calculated and are indicated with an asterisk (e.g., F_ST_*). The values for F_WI_* are indicated by yellow dots in Figure 5A,B, and they are all higher than the corresponding F_WI_ seen with the STAB and MOD subsets with the diploid data (Figure 5B) and also at comparable N’_a_ values in Figure 2B.

## 4. Discussion

AMOVA and SIDTA (Shannon informational diversity translation analysis) are shown to yield highly different assessments of the hierarchical genetic structure of populations, with all levels of the hierarchy being affected. Thus, using either diversity or variance to explore the genetic structure of populations is akin to studying a species using a visual assessment versus using one based on scent. Differences between the two approaches are most pronounced when markers are highly stable and also when hypervariable markers are employed. Furthermore, wrestling with negative among group variance values and F statistic values frequently arises in population studies based on AMOVA, particularly with allopolyploids. This problem is a non-issue with SIDTA, which lacks negative among group differentiation values.

AMOVA also yields the [<0, 1] MDAP-formatted PHiPT index (an estimate of Φ_PT_, an analogue of F_STv_) [27]. PH_i_PT calculates population differentiation simply based on genotypic variance by suppressing variation within individuals. Thus, PhiPT is typically larger than F_STv_ (or more negative) and its behavior as N′_a_ changes closely tracks that of F_STv_. It was excluded from this study because it ignored variance within individuals. A similar [0, 1] index could be created for SIDTA:

Ψ_AP_ =Δ_AP_/(Δ_AP_ + Δ_AI_) (21)

Unlike the heterozygosity- and variance-based MDAP-formatted indices (e.g., F_STv_, F_STh_, G_ST_, G′_ST_N), which measure fixation, the MDAP-formatted Ω_AP_ index yields an assessment of differentiation among populations based on allelic diversity. The addition of Ω_AP_ provides SIDTA with MDAP-, TDAP-, and ENP-based indices. Additionally, the suite of MD-formatted differentiation indices (e.g., Ω_AI_, Ω_AP_, Ω_AR_, etc.) allows for SIDTA-based analyses of the hierarchical genetic structure of populations to be clearly and effectively presented, as shown in Figure 5 and Figure 6.

### 4.1. Formats of Measurement

In addition to distinguishing between indices that measure fixation from those measuring differentiation, it is also useful to group them by how they express this (e.g., focus on the mean difference among populations (MDAP) vs. focus on the total difference among populations (TDAP)). For instance, a recent paper notes that Jost’s D is often mistakenly used as an estimator of G_ST_ [1]. Such a misunderstanding would be less likely if, in addition to knowing that G_ST_ is a fixation index and Jost’s D is a differentiation index, it was also understood that the former has an MDAP format, and the latter has a TDAP format.

### 4.2. Effective Number of What?

It has been shown that expected homozygosity data can be translated into effective number of alleles [2,33]. Although both the q = 1-based SIDTA and the q = 2-based expected heterozygosity measures can be associated with effective numbers data, just what the effective numbers of alleles represents differs between the two approaches. The q = 1 effective number of alleles (exponential of Shannon entropy) is the number of equally common alleles needed to achieve the entropy of allele identity in the population. It is not tied to a given ploidy level and is also not focused on the extent of heterozygosity that is present. In contrast, the q = 2 effective number of alleles is the number of equally common alleles needed to achieve the expected heterozygosity of the alleles in a population of diploid organisms. Thus, D′_AP_ and Jost’s D (and D_EST_) clearly measure different parameters. They only yield the same value when (1) two populations have identical alleles and allele frequencies (D_EST_ = Jost’s D = D′_AP_ = 0.0), and when (2) there are no shared alleles between two populations (D_EST_ = Jost’s D = D′_AP_ = 1.0). Between these two extremes, and based on DS-II and DS-III, D′_AP_ is typically both greater than, and also tracks closer to N′_a_ than either Jost’ D or D_EST_ do (see Figure 3 and Figure 4B).

### 4.3. Comparison with Other among Population Indices

The only MDAP index to measure differentiation examined in this study is Ω_AP_. The other widely used heterozygosity-based and variance-based MDAP indices are all fixation measures. Based on this survey, Ω_AP_ is the sole choice if one needs an assessment of mean differentiation among populations.

The TDAP indices examined in this study all measure allelic differentiation, but they differ in the parameters that they measure: D′_AP_ tracks allelic diversity, the heterozygosity-based indices (G′_ST_H, G″_ST_ (=F′_STv_), Jost’s D, D_EST_) focus on the probability of alleles being in a heterozygous pairing, F′_STv_ measures variance among genotypes. Not a part of the Hill-number family of diversity measures [21,34], BCGD measures allelic differentiation by comparing the similarity of simple allele frequencies (or relative allele frequency) between two populations. Although BCGD was found by [21] to have close a relationship to G_ST_ and F_ST_, this study unequivocally shows that BCGD yields results close to those of the q = 1-based SIDTA D′_AP_ and that they are highly divergent from those of the q = 2 indices (both expected heterozygosity based and variance based).

Although all of the TDAP-formatted indices are equal to 1.0 with DS-I, that is what they were designed to do at the maximum values of the respective parameters that they measure. However, there are striking differences among their overall respective performances in DS-II and DS-III (Figure 3 and Figure 4B). The most notable demarcation among the TDAP indices shows two primary subgroups, with D′_AP_ and BCGD in one group (subclass I) and all of the q = 2 indices in a second grouping (F′_STv_ (=G″_ST_), G′_ST_H, Jost’s D, D_EST_) (subclass II). Subclass I indices track ‘q = 0’ far more closely than the ‘q = 2’ subclass indices do. This reflects how the subclass I indices are related to ‘q = 0’ diversity values, where each allele has the same weight (1). BCGD is based on simple allelic frequency (or relative allele frequency), which is related to q = 0 data (where each allele has equal weighting) by ‘1∙p_i_’, where p_i_ is the relative frequency of an allele in a population. BCGD differs from the q = 1 SIDTA indices, which are related with q = 0 data by ‘1∙p_i_∙(ln p_i_)’. The SIDTA approach tends to dampen (i.e., make more equitable) the differences in frequency among the alleles in a population. Thus, these two subclasses I indices typically remain relatively close to q = 0 diversity data. Although BCGD closely tracks changes in allelic diversity among populations with DS-I and DS-II, it fails to always do so with DS-III. This indicates that BCGD does not track changes in allelic diversity in some cases. In contrast, the SIDTA-based D′_AP_ closely tracks changes in allelic diversity across all three of the stress test data sets.

In contrast, the incorporation of q = 2 data with q = 0 data is achieved by ‘1∙p_i_^2^’, with the squaring of relative frequency data typically greatly increasing the difference between q = 0-based data and q = 2-based data, particularly when compared to that associated with the subclass I indices. Squaring the relative frequency of each allele results in more weight being given to alleles having high frequency and very little weight being given to uncommon alleles. This results in q = 2 indices measuring notably different parameters than those measured by the subclass I indices. The heterozygosity-based TDAP indices, which include Jost’s D, D_EST_, G′_ST_H. and G″_ST_ (=F′_STv_), are unequivocally shown to be tracking changes in the probability of expected heterozygosity by their performance in DS-II and DS-III. Another difference between the two subclasses is that subclass I indices are positive across the three stress test data sets while the adjusted heterozygosity-based indices and the variance-based indices of the subclass II have both positive and negative values.

The two unadjusted heterozygosity indices, F_STh_ and Jost’s D, which are restricted to lie within the interval from 0 to 1, are both positive across all three stress test data sets. In contrast, negative values occur in the ‘adjusted’ expected heterozygosity indices (MDAP: G_ST_, G′_ST_N (=F_STv_); TDAP: G′_ST_H, G″_ST_ (=F_STv_), D_EST_). The ‘adjusted’ expected heterozygosity indices are all positive in DS-1, all negative in DS-II, and both negative and positive with DS-III. The negative values result from the adjustments for small population size and inbreeding in the calculations made by GenAlEx for these indices. The adjustment leads to increases in both H_S_ and H_T_ relative to the corresponding unadjusted H_S_ and H_T_ values, but with H_S_ being increased more than H_T_. The imbalance between the adjusted H_S_ and H_T_ values becomes greater with increases in N′_a_ and negative values for estimated heterozygosity occur when H_S_ > H_T._ With DS-II, this adjustment results in the adjusted estimated heterozygosity values always being negative. The occurrence of negative ‘among population’ values in the ‘adjusted’ heterozygosity indices closely tracks the occurrence of negative values in F_STv_ and F′_STv_. Consequently, one of the notable outcomes of ‘adjustment’ made by GenAlEx to several of the heterozygosity-based indices is that it allows for heterozygosity-based indices to more closely match the entire spectrum of F_STv_ and F’_STv_ based values. Indeed, a bit of additional tweaking results in two heterozygosity-based indices being equivalent to variance-based indices (i.e., G′_ST_N = F_STv_ and G″_ST_ = F′_STv_).

### 4.4. Drawbacks to ‘q = 2’ Indices

Although it is not an issue for q = 1 differentiation measures, one drawback for q = 2-based differentiation measures is that an incremental change in allele number and frequency has a much smaller change on the resulting value of a given index when N′_a_ is lower than when that same incremental change occurs when N′_a_ is very high, given their sigmoidal nature. This is problematic for a differentiation measure to have. Another drawback to the q = 2 differentiation measures is that they were primarily designed for diploid genotypic data. Thus, their application to allopolyploids is problematic, especially with gametophytic data. For instance, negative variance values are considered as representing excess heterozygosity [35] and/or the absence of population structure [36]. However, homologous chromosomes are typically lacking in the gametophytes of allopolyploid plants having disomic inheritance. Thus, ‘true’ heterozygosity does not usually occur in the gametophytes of such allopolyploids. Differences among/between the component monoploid genomes of alloploid gametophytes, which is measured by the allelic diversity within individuals (Δ_WI_), represents the allelic differentiation (Ω_WI_) among/between the ancestral monoploid genomes, not heterozygosity [37]. In such cases, negative F statistics should unequivocally not be considered as having excessive heterozygosity. In terms of negative variance values reflecting a lack of population structure, the data presented here shows that, based on SIDTA, structure is often present in allopolyploid gametophytes when negative variance occurs based on AMOVA. In the absence of population structure, SIDTA would yield ‘0.0’, not a negative value.

### 4.5. Impact of Marker Variability

Marker variability has a major effect on the genetic structure of populations and the nature of this influence differs markedly when based on SIDTA versus AMOVA. Some of these effects are discussed in the section on negative F statistics below.

With both haploids and diploids, the proportion of total allelic diversity within individuals (Ω_WI_) decreases with increasing marker variability while the reverse is the case for the proportion of variance within individuals (F_WI_). This pattern also holds true for Ω_WI_ in allopolyploids, and, in the absence of negative values, it is also true for F_WI_. However, the frequent occurrence of negative values with AMOVA-based F statistics both greatly confuse this pattern for allopolyploids, and, also, notably distort the genetic structure of a population. The negative values are often associated with, but not limited to, F_AI_ (the proportion of total variance (F_T_) represented by variance among individuals within populations). The most negative values occur with highly stable markers and then decline with increasing marker variability and, in some cases negative F values may be lacking with hypervariable markers. In contrast, negative values are lacking altogether in SIDTA-based indices.

Careful consideration needs to be given to the variability of markers selected to study genetic structure as well as their influence on the method(s) of analysis used. In terms of marker variability, a strong focus on highly variable markers leads to a high degree of divergence among individuals within a stratum, and this has the potential to minimize, or completely mask, strong evolutionary signals that may be found in markers having lower variability [18,19,37]. In addition, the resulting high divergence among individuals within a stratum obtained by highly variable markers yields notably lower overall similarity among its members. As the level of divergence among its individuals increases, the cohesive nature of a species decreases.

### 4.6. Notes on Negative ‘F’ Statistics with AMOVA

Although lacking with SIDTA, negative F statistics are ‘part and parcel’ of AMOVA and are notably particularly an issue with allopolyploids. Negative values notably distort the interpretation of the genetic structure of a population, making interpretation difficult. They are often associated with, but not limited to, F_AI_. As noted above, the most negative ‘F’ values occur with highly stable markers and then decline with increasing marker variability. The following is a brief discussion about the negative ‘F’ statistics associated with allopolyploids.

Based on DS-I, F_WI_ is directly closely tied to N′_a_ (Figure 2A), with high values of F_WI_ only occurring at correspondingly high values of N′_a_. This is not always the case, however, as can be seen with the allopolyploid data where, in addition to the HYPE SSRs, high values of F_WI_* are also associated with the STAB and MOD subsets (Figure 6). In allopolyploids having disomic inheritance, the difference among the component monoploid genomes typically result in a very high a mean diversity and variance within individuals (D_WI_), and these particular values are largely independent of marker variability. For instance, based on SIDTA, D′_WI_ for the allodiploid data is (D′_WI_ ≥ 0.97) across the STAB, MOD, and HYPE subsets, and that for allotriploid data is (D′_WI_ ≥ 0.87). This implies that the corresponding estimated variance within each individual is close to maximum. Furthermore, negative estimated variance among individuals augments both F_WI_ and F_WI_*. Both Ω_AI_ and F_AI_ in contrast, are highly affected by marker variability. In allopolyploids, markers having low to moderate variability (such as the STAB and MOD subsets) frequently yield mean square among individuals within populations values that are less than the corresponding high mean square within individual values with AMOVA, which results in negative F_AI_ values. Markers having a sufficiently high variability (such as the HYPE subset) may yield mean square among individuals within populations values that exceed the high mean square within individual values, and thus provide positive F_AI_ values in allopolyploids, as is the case for the allotriploid data (Figure 6B).

### 4.7. Influences of Ploidy Level

Ploidy level has two major influences in a comparison of AMOVA and SIDTA. One is at the haploid level, where there is allelic diversity within individuals (D_WI_ = 1.0), while there is no variation among individuals (F_WI_ = 0.0) with AMOVA. The second influence occurs primarily at diploid and higher ploidy levels where high divergence occurs among the respective component monoploid genomes. In such cases, AMOVA and the foundationally adjusted heterozygosity measures often yield negative values. Negative among group F values can occur with haploid data, but they occur more frequently at higher ploidy values, particularly with allopolyploids. Although this aspect is primarily a result of the extent of divergence among the component monoploid genomes, it is also influenced by ploidy level. 

### 4.8. Interpreting F_ST_ Data from Prior Studies

Studies misinterpreting F_ST_ (both F_STv_ and F_STh_) and G_ST_ as measuring differentiation have the major problem that their conclusions unequivocally do not measure differentiation. Based on marker variability one can gain some general information to partially mitigate this issue. Generally, but not always, highly stable markers are likely to have F_ST_ and G_ST_ values exceeding the corresponding allelic differentiation between/among populations (Ω_AP_). When highly variable markers are used, there is a high probability that the F_ST_ values are much lower than corresponding Ω_AP_ values. Additionally, when moderately stable markers are used it is possible that their associated F_ST_ values may be somewhat comparable to Ω_AP_, despite measuring different things. The above is based on haploid and diploid ploidy levels. This pattern does not always apply to allopolyploids, where the frequent occurrence of negative F values, particularly F_AI_, distorts the interpretation of population structure.

### 4.9. Possible Issue with Adjustments Made for Small Population Size and Inbreeding

For both the fixation and differentiation heterozygosity-based indices, adjustments for the almost unbiased estimators of heterozygosity [28] and for inbreeding [29] result in the adjusted heterozygosity-based indices (1) typically being smaller than the corresponding unadjusted values (e.g., Jost’s D > D_EST_), and (2) yielding both positive to negative values while the unadjusted heterozygosity-based indices are always positive. However, the adjustments affect these fixation and differentiation indices differently. All three of the stress test data sets show the adjusted heterozygosity-based fixation indices (G_ST_, G′_ST_N) to closely follow F_STh_ across all subsets. In contrast, the heterozygosity-based differentiation indices show a more variable pattern. This is most clearly shown in Figure 4B, where positive D_EST_ (as well as G″_ST_ and G′_ST_H) values closely track Jost’s D when N′_a_ ≤ 0.5, but they greatly diverge from Jost’s D when N′_a_ > 0.5, and ultimately become highly negative. These findings indicate that corrections, such as almost unbiased estimators, that are designed for fixation indices are problematic when applied to differentiation indices, particularly at high levels of allelic diversity. Given that the differentiation and fixation indices greatly differ in what they measure, it is not surprising that this is the case. Exploring why such adjustments result in such a complex relationship between Jost’s D and the adjusted heterozygosity-based differentiation indices (D_EST_, G″_ST_ and G′_ST_H) is beyond the scope of this study.

### 4.10. Final Thoughts

As the various allelic differentiation indices measure notably different parameters, it is clear that one size does not fit all. Thus, it is important to select the index (or indices) that match the focus of research being undertaken. If heterozygosity or variance are important causal variables in the given application, then heterozygosity-based q = 2 differentiation measures (e.g., Jost’s D, D_EST_, F′_ST_) should be used [15]. If alleles should be weighed by their population share, then q = 1 differentiation measures (e.g., D′_AP_, Ω_AP_) would be the clear choice. Finally, if the presence or absence of alleles is what matters, then a q = 0 differentiation measure should be used. In addition, there are other aspects to consider, including: (1) when the focus is on the hierarchical genetic structure of populations and not simply on differentiation among populations; (2) avoidance of the problem of negative values; (3) when studying allopolyploids. For these issues, the q = 1-based SIDTA suite of indices clearly fits the bill.

Because the subclass II (q = 2) differentiation measures (1) can decline with increases in allelic diversity and (2) can be negative, they unequivocally have limitations in the measurement of differentiation. Given this, studies focused primarily on how different two or more groups (e.g., populations, sub-species, species, etc.) are would most likely be best served by subclass I differentiation measures (e.g., BCGD, SIDTA). This is especially true when comparing groups above the population level, where allelic diversity likely plays a bigger role than either heterozygosity or variance do.

## Figures and Tables

**Table 1:** Hierarchical population structure based on SIDTA (adapted from [8]). (EFNA: effective number of alleles). The theoretical minimum D_T_ (D_T-MIN_) occurs when just one allele is present across all populations, and the theoretical maximum D_T_ (D_T_-_MAX_) occurs with no allelic overlap within and among all samples across all populations.

Allelic Diversity within Strata (as Effective Number of Alleles)	Differentiation between Strata [Multiplicative] (as Effective Number of Groups)	
D_T_ = (grand total EFNA)	D_AR_ = (EFN regions within study)	
	D_AR_ = (D_T_/D_WR_)	(3)
D_WR_ = (mean EFNA within regions)	D_AP_ = (EFN populations within regions)	
	D_AP_ = (D_WR_/D_WP_)	(4)
D_WP_ = (mean EFNA within populations)	D_AI_ = (EFN individuals within populations)	
	D_AI_ = (D_WP_/D_WI_)	(5)
D_WI_ = (mean EFNA within individuals)		

**Table 3:** Hierarchical population structure based on the mean allelic diversity (Δ) among groups expressed as a proportion (Ω) of the grand total allelic diversity (D_T_).

Mean [0, 5] Differentiation between Strata	
Ω_AP_: Mean [0–0.5] scaled proportion of D_T_ represented by D_AP_	
Ω_AP_ = Δ_AP_/D_T_	(12)
Ω_AI_: Mean [0–0.5] scaled proportion of D_T_ represented by D_AI_	
Ω_AI_ = Δ_AI_/D_T_	(13)
Ω_AIT_ = Mean [0–0.5] scaled proportion of D_T_ represented by D among all inds.	
Ω_AIT_ = Δ_AIT_/D_T_	(14)
Ω_AIP_ = Mean [0–0.5] scaled proportion of D_WP_ represented by D_AI_	
Ω_AIP_ = Δ_AI_/D_WP_	(15)

## Data Availability

The data sets used in this study can be found in Supplemental Files S1 and S2 of this paper.

## Supplementary Materials

The following supporting information can be downloaded at: https://www.mdpi.com/article/10.3390/e25030492/s1. Supplemental File S1: Artificial data sets. Supplemental File S2: Natural data sets. Table S1. Theoretical maximum and minimum values for allelic diversity and variance. Table S2. Range of values and expected behavior of the statistics used in this paper
