# Supplementary material for: A Comparison of Entropic Diversity and Variance in the Study of Population Structure
Source: Entropy (Basel). 2023 Mar 13;25(3):492. doi: 10.3390/e25030492 (PMC10048111; doi:10.3390/e25030492)
Supplement: Supplementary file 1 [file entropy-25-00492-s001.zip › entropy-1939759-supplementary/File S1 - Artificial populations data files.pdf]

Data Set I

Nine (9) pairs of artificial diploid populations (subsets) having ten samples per population, no allelic overlap between populations, and with equal AMD (D), equal heterozygosity, and equal variance within each population. By design, W_{AP} was 50% and the TDAP formatted indices were at the theoretical maximum for each subset.

Each data set is based on one 'marker'.

Alleles for each sample are pla

N_a = total # alleles per subset

$N_{a\text{-MAX}}$ = theoretical maximum N_a per subset $N_{a\text{-MAX}} = 40$

N'_a = N_a expressed as a proportion of $N_{a\text{-MAX}}$

Data subsets

		**SS-.9**		**SS-.8**		**SS-.7**		**SS-.6**		**SS-.5**		**SS-.4**		**SS-.3**		**SS-.2**		**SS-.1**	
										**N'a**									
Sample	Population	0.9		0.8		0.7		0.6		0.5		0.4		0.3		0.2		0.1	
1	A	1	1	1	1	1	1	1	1	1	1	1	1	1	1	1	1	1	1
2	A	1	12	1	1	1	1	1	1	1	1	1	1	1	1	1	1	1	1
3	A	3	13	1	13	1	1	1	1	1	1	1	1	1	1	1	1	1	1
4	A	4	14	4	14	1	14	1	1	1	1	1	1	1	1	1	1	1	1
5	A	5	15	5	15	5	15	1	15	1	1	1	1	1	1	1	1	1	1
6	A	6	16	6	16	6	16	6	16	1	16	1	1	1	1	1	1	1	1
7	A	7	17	7	17	7	17	7	17	7	17	1	17	1	1	1	1	1	1
8	A	8	18	8	18	8	18	8	18	8	18	8	18	1	18	1	1	1	1
9	A	9	19	9	19	9	19	9	19	9	19	9	19	9	19	1	19	1	1
10	A	10	20	10	20	10	20	10	20	10	20	10	20	10	20	10	20	1	20
11	B	51	51	51	51	51	51	51	51	51	51	51	51	51	51	51	51	51	51
12	B	51	62	51	51	51	51	51	51	51	51	51	51	51	51	51	51	51	51
13	B	53	63	51	63	51	51	51	51	51	51	51	51	51	51	51	51	51	51
14	B	54	64	54	64	51	64	51	51	51	51	51	51	51	51	51	51	51	51
15	B	55	65	55	65	55	65	51	65	51	51	51	51	51	51	51	51	51	51
16	B	56	66	56	66	56	66	56	66	51	66	51	51	51	51	51	51	51	51
17	B	57	67	57	67	57	67	57	67	57	67	51	67	51	51	51	51	51	51
18	B	58	68	58	68	58	68	58	68	58	68	58	68	51	68	51	51	51	51
19	B	59	69	59	69	59	69	59	69	59	69	59	69	59	69	51	69	51	51
20	B	60	70	60	70	60	70	60	70	60	70	60	70	60	70	60	70	51	70

		**SS-.05**		**Min SS**		**Max SS**	
Sample	Population	0.05		0.025		1	
1	A	1	1	1	1	1	11
2	A	1	1	1	1	2	12
3	A	1	1	1	1	3	13
4	A	1	1	1	1	4	14
5	A	1	1	1	1	5	15
6	A	1	1	1	1	6	16
7	A	1	1	1	1	7	17
8	A	1	1	1	1	8	18
9	A	1	1	1	1	9	19
10	A	1	1	1	1	10	20
11	B	51	51	1	1	51	61
12	B	51	51	1	1	52	62
13	B	51	51	1	1	53	63
14	B	51	51	1	1	54	64
15	B	51	51	1	1	55	65
16	B	51	51	1	1	56	66
17	B	51	51	1	1	57	67
18	B	51	51	1	1	58	68
19	B	51	51	1	1	59	69
20	B	51	51	1	1	60	70

Data Set II

Ten (10) pairs of artificial populations (subsets) having the same parameters as the subsets in Data Set I, with the exceptions of (1) allelic overlap occurring between the two populations in each subset and (2) TDAP indices were not all at the theoretical maximum.

N_a = total # alleles per subset

$N_{a\text{-}MAX}$ = theoretical maximum N_a per subset $N_{a\text{-}MAX} = 40$

$N'_a = N_a$ expressed as a proportion of $N_{a\text{-}MAX}$

Each data set is based on one 'marker'.

Alleles for each sample are placed in columns

Data subsets

		**SS-.93**		**SS-.88**		**SS-.78**		**SS-.68**		**SS-.58**		**SS-.48**		**SS-.38**		**SS-.28**		**SS-.18**		**SS-.08**		**SS-.05**		**Min SS**		**Max SS**	
												**N'_a**															
Sample	Populatior	0.925		0.875		0.775		0.675		0..575		0.475		0.375		0.275		0.175		0.075		0.05		0.025		1	
1	a	1	11	1	1	1	1	1	1	1	1	1	1	1	1	1	1	1	1	1	1	1	1	1	1	1	11
2	a	2	12	1	12	1	1	1	1	1	1	1	1	1	1	1	1	1	1	1	1	1	1	1	1	2	12
3	a	3	13	3	13	1	13	1	1	1	1	1	1	1	1	1	1	1	1	1	1	1	1	1	1	3	13
4	a	4	14	4	14	4	14	1	14	1	1	1	1	1	1	1	1	1	1	1	1	1	1	1	1	4	14
5	a	5	15	5	15	5	15	5	15	1	15	1	1	1	1	1	1	1	1	1	1	1	1	1	1	5	15
6	a	6	16	6	16	6	16	6	16	6	16	1	16	1	1	1	1	1	1	1	1	1	1	1	1	6	16
7	a	7	17	7	17	7	17	7	17	7	17	7	17	1	17	1	1	1	1	1	1	1	1	1	1	7	17
8	a	8	18	8	18	8	18	8	18	8	18	8	18	8	18	1	18	1	1	1	1	1	1	1	1	8	18
9	a	9	19	9	19	9	19	9	19	9	19	9	19	9	19	9	19	1	19	1	1	1	1	1	1	9	19
10	a	10	20	10	20	10	20	10	20	10	20	10	20	10	20	10	20	10	20	1	20	1	1	1	1	10	20
11	b	1	1	1	1	1	1	1	1	1	1	1	1	1	1	1	1	1	1	1	1	51	51	1	1	51	61
12	b	1	62	1	62	1	1	1	1	1	1	1	1	1	1	1	1	1	1	1	1	51	51	1	1	52	62
13	b	53	63	53	63	1	63	1	1	1	1	1	1	1	1	1	1	1	1	1	1	51	51	1	1	53	63
14	b	54	64	54	64	54	64	1	64	1	1	1	1	1	1	1	1	1	1	1	1	51	51	1	1	54	64
15	b	55	65	55	65	55	65	55	65	1	65	1	1	1	1	1	1	1	1	1	1	51	51	1	1	55	65
16	b	56	66	56	66	56	66	56	66	56	66	1	66	1	1	1	1	1	1	1	1	51	51	1	1	56	66
17	b	57	67	57	67	57	67	57	67	57	67	57	67	1	67	1	1	1	1	1	1	51	51	1	1	57	67
18	b	58	68	58	68	58	68	58	68	58	68	58	68	58	68	1	68	1	1	1	1	51	51	1	1	58	68
19	b	59	69	59	69	59	69	59	69	59	69	59	69	59	69	59	69	1	69	1	1	51	51	1	1	59	69
20	b	60	70	60	70	60	70	60	70	60	70	60	70	60	70	60	70	60	70	1	70	51	51	1	1	60	70

Eric F. Karlin q = 1’ based indices measure allelic differentiation, ‘q = 2’ based indices don’t

Data Set III

Ten (10) pairs of artificial populations (subsets) were created based on the same parameters used for the subsets in DS-II except that heterozygosity, variance, and Δ are not balanced between the two populations in each subset.

N_a = total # alleles per subset

$N_{a\text{-}MAX}$ = theoretical maximum N_a per subset $N_{a\text{-}MAX} = 40$

N'_a = N_a expressed as a proportion of $N_{a\text{-}MAX}$

Each data set is based on one 'marker'.

Alleles for each sample are placed in columns

Data subsets 0.025

		**SS-.9**		**SS-.8**		**SS-.7**		**SS-.6**		**SS-.5**		**SS-.4**		**SS-.3**		**SS-.2**		**SS-.1**		**SS-.05**		**Min SS**		**Max SS**	
												**N'a**													
		0.9		0.8		0.7		0.6		0.5		0.4		0.3		0.2		0.1		0.05		0.025		1.0	
1	a	1	1	1	1	1	1	1	1	1	1	1	1	1	1	1	1	1	1	1	1	1	1	1	11
2	a	1	12	1	1	1	1	1	1	1	1	1	1	1	1	1	1	1	1	1	1	1	1	2	12
3	a	3	13	1	1	1	1	1	1	1	1	1	1	1	1	1	1	1	1	1	1	1	1	3	13
4	a	4	14	1	14	1	1	1	1	1	1	1	1	1	1	1	1	1	1	1	1	1	1	4	14
5	a	5	15	5	15	1	1	1	1	1	1	1	1	1	1	1	1	1	1	1	1	1	1	5	15
6	a	6	16	6	16	1	16	1	1	1	1	1	1	1	1	1	1	1	1	1	1	1	1	6	16
7	a	7	17	7	17	7	17	1	1	1	1	1	1	1	1	1	1	1	1	1	1	1	1	7	17
8	a	8	18	8	18	8	18	1	18	1	1	1	1	1	1	1	1	1	1	1	1	1	1	8	18
9	a	9	19	9	19	9	19	9	19	1	1	1	1	1	1	1	1	1	1	1	1	1	1	9	19
10	a	10	20	10	20	10	20	10	20	1	20	1	1	1	1	1	1	1	1	1	1	1	1	10	20
11	b	1	1	1	1	1	1	1	1	1	1	1	1	1	1	1	1	1	1	1	1	1	1	51	61
12	b	52	62	52	62	52	62	52	62	63	62	1	1	1	1	1	1	1	1	1	1	1	1	52	62
13	b	53	63	53	63	53	63	53	63	53	63	1	63	1	1	1	1	1	1	1	1	1	1	53	63
14	b	54	64	54	64	54	64	54	64	54	64	54	64	1	1	1	1	1	1	1	1	1	1	54	64
15	b	55	65	55	65	55	65	55	65	55	65	55	65	1	65	1	1	1	1	1	1	1	1	55	65
16	b	56	66	56	66	56	66	56	66	56	66	56	66	56	66	1	1	1	1	1	1	1	1	56	66
17	b	57	67	57	67	57	67	57	67	57	67	57	67	57	67	1	67	1	1	1	1	1	1	57	67
18	b	58	68	58	68	58	68	58	68	58	68	58	68	58	68	58	68	1	1	1	1	1	1	58	68
19	b	59	69	59	69	59	69	59	69	59	69	59	69	59	69	59	69	1	69	1	1	1	1	59	69
20	b	60	70	60	70	60	70	60	70	60	70	60	70	60	70	60	70	60	70	1	70	1	1	60	70
