# Supplementary material for: A Comparison of Entropic Diversity and Variance in the Study of Population Structure
Source: Entropy (Basel). 2023 Mar 13;25(3):492. doi: 10.3390/e25030492 (PMC10048111; doi:10.3390/e25030492)
Supplement: Supplementary file 1 [file entropy-25-00492-s001.zip › entropy-1939759-supplementary/File S2 - natural populations data files.pdf]

Eric F. Karlin A comparison of diversity and variance in the study of genetic structure of populations

Data adapted from: Karlin, E.F., Boles, S.B.; Shaw, A.J. Resolving boundaries between species in Sphagnum section Subsecunda using microsatellite markers. Taxon 2008 57, 1189-1200. doi.org/10.1002/tax.574012

Haploid data

Three data sets for regional populations of haploid gametophytes of *Sphagnum comosum* Müll. Hal. and *S. novo-zelandicum* Mitt. (14 samples each).

		STAB SSRs											MOD SSRs			HYPE SSRs	
Sample	Taxon	**22**	**17**	**1**	**5**	**19**	**18**	**16**	**29**	**28**	**9**	<SSR	**4**	**12**	**20**	**10**	**14**
EK4	*S. comosum*	102	158	254	192	252	141	254	190	240	180		186	123	297	225	239
EK55	*S. comosum*	102	158	249	192	252	141	254	190	240	180		186	123	297	225	237
EK56	*S. comosum*	102	158	249	192	252	141	254	190	240	180		184	123	294	225	237
EK8	*S. comosum*	108	158	254	192	252	138	254	190	238	180		186	123	291	229	245
EK27	*S. comosum*	108	158	249	192	252	141	254	190	240	180		184	123	297	233	219
EK229a	*S. comosum*	112	158	255	192	252	138	254	190	240	180		184	123	291	243	237
SB1832	*S. comosum*	108	158	254	192	252	138	254	190	238	164		184	123	297	239	219
SB1833	*S. comosum*	102	158	249	192	252	138	254	190	238	182		184	123	294	225	239
SB127	*S. comosum*	102	158	254	192	252	138	254	190	240	180		184	123	297	241	219
SB1132	*S. comosum*	102	158	254	192	252	138	254	190	240	180		184	123	297	241	219
EK326	*S. comosum*	102	158	254	193	252	141	254	190	240	180		186	123	297	241	219
EK325	*S. comosum*	102	161	268	192	252	138	254	190	242	180		186	123	297	223	233
EK324	*S. comosum*	102	158	254	192	252	138	254	190	240	180		186	123	297	227	241
EK322f	*S. comosum*	102	158	249	192	252	138	254	190	238	180		184	123	294	243	239
SB881	*S. novo-zelandicum*	108	158	254	192	252	138	254	202	238	180		197	130	288	233	235
EK3	*S. novo-zelandicum*	105	164	254	194	252	138	254	202	238	180		195	132	288	237	229
EK60	*S. novo-zelandicum*	108	164	254	194	252	132	254	202	238	180		195	132	291	231	229
EK59	*S. novo-zelandicum*	108	158	254	194	252	132	254	202	238	180		175	132	288	241	221
EK62	*S. novo-zelandicum*	108	158	254	194	252	132	254	202	238	180		191	128	288	247	219
EK14	*S. novo-zelandicum*	108	161	255	194	252	141	254	202	238	180		184	132	288	237	211
EK34	*S. novo-zelandicum*	108	161	255	194	252	141	254	202	238	180		195	132	288	237	211
EK15	*S. novo-zelandicum*	108	161	255	194	252	141	254	202	238	180		195	124	288	239	211
EK327n	*S. novo-zelandicum*	102	167	254	194	252	138	254	202	238	180		193	135	288	235	227
EK224ci	*S. novo-zelandicum*	108	158	254	194	252	138	254	202	238	180		195	128	282	239	223
EK227a	*S. novo-zelandicum*	108	164	254	194	252	138	254	202	238	180		195	132	288	243	225
EK228a	*S. novo-zelandicum*	108	164	254	194	252	138	254	202	238	180		184	128	288	241	231
EK230a	*S. novo-zelandicum*	108	164	254	194	252	138	254	202	238	180		195	132	288	239	219
SB1136a	*S. novo-zelandicum*	108	158	254	192	252	138	254	202	238	180		195	128	285	245	217

Eric F. Karlin A comparison of diversity and variance in the study of genetic structure of populations

Data adapted from: Karlin, E.F., Boles, S.B.; Shaw, A.J. Resolving boundaries between species in *Sphagnum* section *Subsecunda* using microsatellite markers. *Taxon* 2008 57, 1189-1200.

Diploid data

Three data sets for two 'artificial' diploid sporophyte populations of *Sphagnum novo-zelandicum Mitt.* (6 diploid samples per population)

Alleles for each SSR are placed in rows (not in columns)

Haplotype			STAB SSRs			MOD SSRs				HYPE SSRs	
Sample	Genotype↓	SSR>	**22**	**20**	**1**	18	4	12	**17**	10	14
SB881	a		108	288	254	138	197	130	158	233	235
EK3	a		105	288	254	138	195	132	164	237	229
EK60	b		108	291	254	132	195	132	164	231	229
EK59	b		108	288	254	132	175	132	158	241	221
EK62	c		108	288	254	132	191	128	158	247	219
EK14	c		108	288	255	141	184	132	161	237	211
EK327n	d		102	288	254	138	193	135	167	235	227
EK224ci	d		108	282	254	138	195	128	158	239	223
EK227a	e		108	288	254	138	195	132	164	243	225
EK228a	e		108	288	254	138	184	128	164	241	231
EK230a	f		108	288	254	138	195	132	164	239	219
SB1136a	f		108	285	254	138	195	128	158	245	217
EK526	g		108	288	254	138	197	130	158	233	231
EK13	g		108	288	255	141	195	132	161	239	211
SB881	h		108	288	254	138	197	130	158	233	235
EK59	h		108	288	254	132	175	132	158	241	221
EK34	i		108	288	255	141	195	132	161	237	211
EK34	i		108	288	255	141	195	132	161	237	211
EK34	j		108	288	255	141	195	132	161	237	211
EK34	j		108	288	255	141	195	132	161	237	211
EK15	k		108	288	255	141	195	124	161	239	211
EK15	k		108	288	255	141	195	124	161	239	211
EK15	m		108	288	255	141	195	124	161	239	211
EK15	m		108	288	255	141	195	124	161	239	211

Data adapted from: Karlin, E.F., Boles, S.B.; Shaw, A.J. Systematics of *Sphagnum* section *Sphagnum* in New Zealand: a microsatellite-based analysis. New Zealand Journal of Botany 2008, 46, 105–118.
Karlin, E.F.; Hotchkiss, S.C.; *et al.* High genetic diversity in a remote island population system: sans sex. *New Phytologist* **2012** *193* , 1088–1097.

Allodiploid data

Three SSR data sets for regional population of two gametophytically allodiploid *Sphagnum* species (31 samples per population)
Alleles for each SSR are placed in rows (not in columns)

		STAB SSRs										MOD SSRs			HYPE SSRs	
Taxon	Sample↓	22	30	18	5	19	29	1	9	20	<SSR	7	28	4	10	14
S. palustre	42	102	143	132	192	260	192	246	179	292		169	233	199	233	249
S. palustre	42	106	143	135	198	260	196	254	179	292		175	236	199	248	249
S. palustre	34	102	143	132	192	260	192	246	179	292		169	233	199	235	239
S. palustre	34	106	143	135	198	260	196	254	179	292		179	233	199	248	239
S. palustre	37	102	143	132	192	260	192	246	179	292		169	233	199	239	208
S. palustre	37	106	143	135	198	260	196	254	179	292		175	236	199	244	229
S. palustre	24	102	143	132	192	260	192	246	179	292		169	233	199	239	223
S. palustre	24	106	143	135	198	260	196	246	179	292		175	236	199	246	223
S. palustre	36	102	143	132	192	260	192	246	179	292		169	233	199	239	223
S. palustre	36	106	143	135	198	260	196	254	179	292		175	236	199	246	223
S. palustre	38	102	143	132	192	260	192	246	179	292		169	233	199	239	223
S. palustre	38	106	143	135	198	260	196	254	179	292		175	236	199	246	223
S. palustre	39	102	143	132	192	260	192	246	179	292		169	233	199	243	231
S. palustre	39	106	143	135	198	260	196	254	179	292		175	236	199	248	231
S. palustre	41	102	143	132	192	260	192	246	179	292		169	233	199	239	223
S. palustre	41	106	143	135	198	260	196	254	179	292		175	236	199	246	223
S. palustre	94	102	143	132	192	260	192	246	179	292		169	233	199	239	223
S. palustre	94	106	143	135	198	260	196	254	179	292		175	236	199	246	223
S. palustre	25	102	143	132	192	260	192	246	179	292		169	233	199	241	223
S. palustre	25	106	143	135	198	260	196	254	179	292		175	236	199	246	223
S. palustre	17	102	143	132	192	260	192	246	179	292		169	233	199	243	253
S. palustre	17	106	143	135	198	260	196	254	179	292		175	236	199	248	253
S. palustre	95	102	143	132	192	260	192	246	181	292		169	233	199	233	225
S. palustre	95	106	143	135	200	260	196	254	181	292		175	236	199	246	225
S. palustre	2	102	143	132	192	260	192	246	181	292		169	233	200	233	229
S. palustre	2	106	143	135	200	260	196	254	181	292		175	236	200	246	229
S. palustre	92	102	143	132	192	260	192	246	179	284		169	233	199	243	257
S. palustre	92	106	143	135	198	260	196	254	179	284		175	236	199	246	257
S. palustre	18	102	143	135	192	260	192	246	181	292		169	233	199	221	233
S. palustre	18	106	143	138	200	260	196	254	181	292		175	236	199	248	233
S. palustre	20	102	143	135	192	260	192	246	181	292		169	233	199	223	251
S. palustre	20	106	143	138	200	260	196	254	181	292		175	236	199	246	251
S. palustre	21	102	143	135	192	260	192	246	181	292		169	233	199	223	249
S. palustre	21	106	143	138	200	260	196	254	181	292		175	236	199	246	249
S. palustre	22	102	143	135	192	260	192	246	181	292		169	233	199	221	239
S. palustre	22	106	143	138	200	260	196	254	181	292		175	236	199	248	239
S. palustre	56	102	143	132	192	260	192	246	179	284		169	233	199	241	257
S. palustre	56	106	143	135	198	260	196	254	179	284		175	236	199	246	257
S. palustre	57	102	143	132	192	260	192	246	179	284		169	233	199	241	259
S. palustre	57	106	143	135	198	260	196	254	179	284		175	236	199	246	259
S. palustre	58	102	143	132	192	260	192	246	179	284		169	233	199	241	257
S. palustre	58	106	143	135	198	260	196	254	179	284		175	236	199	246	257
S. palustre	59	102	143	132	192	260	192	246	179	284		169	233	200	241	257
S. palustre	59	106	143	135	198	260	196	254	179	284		175	236	200	246	257
S. palustre	60	102	143	132	192	260	192	246	179	284		169	233	199	241	257
S. palustre	60	106	143	135	198	260	196	254	179	284		175	236	199	246	257
S. palustre	61	102	143	132	192	260	192	246	179	292		169	233	199	239	245
S. palustre	61	106	143	135	198	260	196	254	179	292		175	236	199	248	245
S. palustre	62	102	143	132	192	260	192	246	179	292		169	233	199	239	245
S. palustre	62	106	143	135	198	260	196	254	179	292		175	236	199	248	245
S. palustre	63	102	143	132	192	260	192	246	179	292		169	233	200	239	245
S. palustre	63	106	143	135	198	260	196	254	179	292		175	236	200	248	245
S. palustre	64	102	143	132	192	260	192	246	179	284		169	233	199	241	257
S. palustre	64	106	143	135	198	260	196	254	179	284		175	236	199	246	257
S. palustre	65	102	143	132	192	260	192	246	179	292		169	233	200	239	245
S. palustre	65	106	143	135	198	260	196	254	179	292		175	236	200	248	245
S. palustre	66	102	143	132	192	260	192	246	179	284		169	233	199	241	257
S. palustre	66	106	143	135	198	260	196	254	179	284		175	236	199	246	257
S. palustre	67	102	143	132	192	260	192	246	179	292		169	233	199	239	245
S. palustre	67	106	143	135	198	260	196	254	179	292		175	236	199	248	245
S. palustre	68	102	143	132	192	260	192	246	179	284		169	233	199	241	257
S. palustre	68	106	143	135	198	260	196	254	179	284		175	236	199	246	257
S. cristatum	82	86	143	132	196	263	192	246	179	292		170	234	187	231	257
S. cristatum	82	102	139	135	198	263	196	253	179	292		176	238	187	244	257
S. cristatum	83	86	143	132	196	263	196	246	179	292		170	234	187	231	262
S. cristatum	83	102	139	135	198	263	196	253	179	292		176	238	187	244	262
S. cristatum	84	86	143	132	196	263	196	246	179	292		170	234	187	251	237
S. cristatum	84	102	153	135	190	263	196	253	179	292		176	238	187	244	237
S. cristatum	85	86	143	132	196	263	192	246	179	292		170	234	187	231	262
S. cristatum	85	102	139	135	198	263	196	253	179	292		176	238	187	244	262
S. cristatum	86	86	146	132	196	263	196	246	179	295		170	234	187	244	229
S. cristatum	86	102	153	135	198	263	196	253	179	295		176	238	187	244	229
S. cristatum	87	86	143	132	196	263	196	246	179	295		170	234	187	229	241
S. cristatum	87	102	153	135	200	263	196	253	179	295		176	238	187	244	241
S. cristatum	88	86	143	132	196	263	192	246	179	292		170	234	187	233	251
S. cristatum	88	102	153	135	198	263	196	253	179	292		176	238	187	244	251
S. cristatum	90	86	143	132	196	263	192	246	179	292		170	234	187	221	245
S. cristatum	90	102	156	135	198	263	196	253	179	292		176	238	187	244	245
S. cristatum	91	86	143	132	196	263	192	246	179	292		170	234	187	221	247
S. cristatum	91	102	136	135	200	263	196	253	179	292		176	238	187	244	247
S. cristatum	92	86	143	132	198	263	192	246	179	292		170	234	187	221	247
S. cristatum	92	102	153	135	198	263	196	253	179	292		176	238	187	244	247
S. cristatum	93	86	146	132	198	263	192	246	179	292		170	234	187	223	255
S. cristatum	93	102	153	135	200	263	196	253	179	292		176	238	187	244	255
S. cristatum	94	86	143	132	196	263	192	246	179	295		170	234	187	229	260
S. cristatum	94	102	153	135	198	263	196	253	179	295		176	238	187	244	260
S. cristatum	190	86	143	132	196	263	192	246	179	292		170	234	187	246	243
S. cristatum	190	102	153	135	198	263	196	253	179	292		176	238	187	246	243
S. cristatum	233	86	143	132	196	263	192	246	179	292		170	234	187	227	253
S. cristatum	233	102	153	135	198	263	196	253	179	292		176	238	187	246	253
S. cristatum	234	86	143	132	196	263	192	246	179	292		170	234	187	227	235
S. cristatum	234	102	153	135	198	263	196	253	179	292		176	238	187	244	235
S. cristatum	235	86	143	132	196	263	192	246	179	292		170	234	187	227	247
S. cristatum	235	102	153	135	200	263	196	253	179	292		176	238	187	244	247
S. cristatum	238	86	143	132	196	263	192	246	179	292		170	234	187	227	243
S. cristatum	238	102	153	135	198	263	196	253	179	292		174	238	187	244	243
S. cristatum	239	86	146	132	196	263	192	246	179	292		170	234	187	227	233
S. cristatum	239	102	153	135	198	263	196	253	179	292		176	238	187	246	233
S. cristatum	240	86	143	132	196	263	192	246	179	292		170	234	187	247	262
S. cristatum	240	102	153	135	202	263	196	253	179	292		178	238	187	244	262
S. cristatum	242	86	143	132	196	263	192	246	179	292		170	234	187	225	255
S. cristatum	242	102	153	135	198	263	196	253	179	292		178	238	187	242	255
S. cristatum	244	86	143	132	196	263	192	246	179	292		170	234	187	246	231
S. cristatum	244	102	153	135	198	263	196	253	179	292		176	238	187	246	231
S. cristatum	246	86	143	132	198	263	192	246	179	292		170	238	187	231	231
S. cristatum	246	102	153	135	198	263	196	253	179	292		176	238	187	244	231
S. cristatum	248	86	143	132	196	263	192	246	179	292		170	234	187	221	247
S. cristatum	248	102	136	135	200	263	196	253	179	292		176	238	187	244	247
S. cristatum	249	86	143	132	196	263	192	246	179	298		170	234	187	221	265
S. cristatum	249	102	153	135	198	263	196	253	179	298		176	238	187	244	265
S. cristatum	253	86	143	132	196	263	192	246	179	292		170	234	187	227	253
S. cristatum	253	102	153	135	200	263	196	253	179	292		176	238	187	244	253
S. cristatum	1819	86	143	132	196	263	192	246	179	292		170	234	187	233	233
S. cristatum	1819	102	153	135	198	263	196	253	179	292		176	238	187	244	233
S. cristatum	EK320	86	147	132	196	263	192	246	179	292		170	234	187	231	247
S. cristatum	EK320	102	153	135	198	263	196	253	179	292		176	238	187	244	247
S. cristatum	EK321	86	147	132	196	263	192	246	179	292		170	234	187	231	213
S. cristatum	EK321	102	153	135	198	263	196	253	179	292		176	238	187	244	213
S. cristatum	EK322	86	147	132	196	263	192	246	179	292		170	234	187	227	251
S. cristatum	EK322	102	153	135	198	263	196	253	179	292		176	238	187	246	251
S. cristatum	EK325	86	143	132	196	263	192	246	179	292		170	234	187	227	255
S. cristatum	EK325	102	153	135	198	263	196	254	179	292		176	236	187	246	255
S. cristatum	EK326	86	143	132	196	263	192	246	181	292		170	234	187	221	263
S. cristatum	EK326	102	153	135	200	263	196	254	181	292		176	236	187	244	263

Eric F. Karlin A comparison of diversity and variance in the study of genetic structure of populations

Data adapted from: Karlin, E.F.; Smouse, P.E. Holantarctic diversity varies widely among genetic loci within the gametophytically allotriploid peat moss *Sphagnum ×falcatulum*.
American Journal of Botany 2019, 106, 137–144.

Allotriploid data

Three SSR data sets for two population of the gametophytically allotriploid *Sphagnum ×falcatulum* Besch. (21 samples per population)

Alleles for each SSR are placed in three rows (not in columns)

		STAB SSRs										MOD SSRs			HYPE SSRs	
Population	Sample↓	22	1	19	7	4	17	12	18	30	<SSR	9	5	20	10	14
Population A	199	83	259	263	191	186	164	116	132	140		187	194	287	245	229
Population A	199	89	255	257	177	178	164	116	132	143		187	209	293	253	268
Population A	199	112	254	252	179	186	161	121	135	143		176	492	302	308	229
Population A	205	83	259	263	191	186	164	116	132	140		187	194	287	255	227
Population A	205	89	250	257	177	178	164	116	132	143		187	206	290	255	277
Population A	205	112	254	252	179	184	161	123	145	143		198	486	298	364	227
Population A	149	83	259	263	191	182	164	116	132	140		187	194	287	257	231
Population A	149	89	250	257	183	178	164	116	132	143		192	206	284	275	251
Population A	149	112	254	252	179	184	161	121	145	143		194	486	296	294	269
Population A	EK266	83	259	263	191	186	164	116	132	140		187	194	287	217	243
Population A	EK266	89	250	257	181	178	164	116	132	143		187	204	290	273	243
Population A	EK266	112	254	252	179	186	161	121	145	143		194	483	302	321	243
Population A	141	83	259	263	191	186	164	116	132	140		187	200	287	253	235
Population A	141	89	250	257	179	178	164	116	132	143		180	206	290	253	268
Population A	141	112	254	252	179	186	161	121	145	143		190	487	298	341	235
Population A	Ek330	83	259	263	191	186	164	116	132	140		187	200	287	253	227
Population A	Ek330	89	250	257	179	178	164	116	132	143		180	206	290	257	260
Population A	Ek330	112	254	252	179	186	161	121	145	143		190	487	298	337	227
Population A	203	83	259	263	193	186	164	116	132	140		187	194	287	245	247
Population A	203	89	250	257	181	178	164	116	132	143		187	206	290	257	286
Population A	203	112	254	252	179	186	161	121	145	143		209	486	296	345	247
Population A	144	83	259	263	191	186	164	116	132	140		187	194	287	237	233
Population A	144	89	250	257	177	178	164	116	132	143		187	209	290	253	257
Population A	144	112	254	252	179	186	161	121	145	143		200	492	298	368	233
Population A	200	83	259	263	193	186	164	116	132	140		187	194	287	245	249
Population A	200	89	250	257	181	178	164	116	132	143		187	206	290	257	288
Population A	200	112	254	252	179	186	161	121	145	143		217	486	296	343	249
Population A	21	83	259	263	191	186	164	116	132	140		187	194	287	251	233
Population A	21	89	250	257	183	178	164	116	132	143		191	206	284	266	257
Population A	21	112	254	252	179	186	161	121	145	143		195	486	296	308	281
Population A	107	83	259	263	191	186	164	116	132	140		187	194	287	253	251
Population A	107	89	255	257	181	178	164	116	132	143		209	196	281	266	257
Population A	107	112	254	252	179	182	161	121	135	143		217	475	298	321	251
Population A	57	83	259	263	191	186	164	116	132	140		187	194	287	253	251
Population A	57	89	255	257	181	178	164	116	132	143		209	196	281	266	257
Population A	57	112	254	252	179	186	161	121	135	143		217	475	298	321	251
Population A	111	83	259	263	191	186	164	116	132	140		187	194	287	279	262
Population A	111	89	250	257	181	178	167	116	132	143		192	206	298	290	268
Population A	111	115	254	252	179	186	161	121	145	143		194	489	298	368	262
Population A	110	83	259	263	191	186	164	116	132	140		187	194	287	279	262
Population A	110	89	250	257	181	178	167	116	132	143		192	206	298	292	270
Population A	110	115	254	252	179	190	161	121	145	143		194	489	298	368	262
Population A	112	83	259	263	191	186	164	116	132	140		187	194	287	281	264
Population A	112	89	250	257	181	178	167	116	132	143		192	206	298	290	268
Population A	112	115	254	252	179	190	161	121	145	143		194	489	298	368	264
Population A	118	83	259	263	191	186	164	116	132	140		187	194	287	272	260
Population A	118	89	250	257	181	178	167	116	132	143		192	206	298	272	255
Population A	118	115	254	252	179	190	161	121	145	143		194	489	298	272	260
Population A	120	83	259	263	191	186	164	116	132	140		187	194	287	272	260
Population A	120	89	250	257	181	178	167	116	132	143		192	206	298	272	255
Population A	120	115	254	252	179	190	161	121	145	143		194	489	298	272	249
Population A	32sbf	83	259	263	191	186	164	116	132	140		187	194	287	241	253
Population A	32sbf	89	250	257	181	178	164	116	132	143		180	211	293	266	273
Population A	32sbf	115	254	252	179	182	161	121	135	143		205	492	298	364	253
Population A	119f	83	259	263	195	186	164	116	132	140		187	200	287	253	225
Population A	119f	89	255	257	183	178	164	116	132	143		180	206	293	253	255
Population A	119f	112	254	252	179	188	161	121	135	143		209	487	298	323	260
Population A	201f	83	259	263	191	186	164	116	132	140		187	200	287	253	225
Population A	201f	89	255	257	183	178	164	116	132	143		180	206	293	253	255
Population A	201f	112	254	252	179	188	161	121	135	143		209	487	298	323	260
Population A	EK910*	83	259	263	191	186	164	116	132	140		187	194	287	239	233
Population A	EK910*	89	250	257	177	178	164	116	132	143		180	204	296	253	257
Population A	EK910*	112	254	252	179	184	161	123	145	143		190	483	298	255	233
Population B	702	83	259	263	191	186	161	116	132	140		187	194	287	247	227
Population B	702	89	250	257	177	178	164	116	132	143		176	204	296	257	247
Population B	702	105	254	252	179	186	164	123	145	143		180	484	308	345	282
Population B	703	83	259	263	191	186	161	116	132	140		187	194	287	247	227
Population B	703	89	250	257	177	178	164	116	132	143		176	204	296	257	247
Population B	703	105	254	252	179	186	164	123	145	143		180	484	308	345	288
Population B	705	83	259	263	191	186	161	116	132	140		187	194	287	249	227
Population B	705	89	250	257	177	178	164	116	132	143		176	204	296	257	251
Population B	705	105	254	252	179	186	164	123	145	143		180	484	308	347	288
Population B	707	83	259	263	191	186	161	116	132	140		187	194	287	247	227
Population B	707	89	250	257	177	178	164	116	132	143		176	204	296	257	247
Population B	707	105	254	252	179	186	164	123	145	143		180	484	308	345	290
Population B	708	83	259	263	191	186	161	116	132	140		187	200	287	255	245
Population B	708	89	250	257	183	178	164	116	132	143		200	206	284	275	256
Population B	708	112	254	252	179	182	164	121	145	143		200	488	305	331	271
Population B	709	83	259	263	191	186	161	116	132	140		187	200	287	255	245
Population B	709	89	250	257	183	178	164	116	132	143		198	206	284	275	256
Population B	709	112	254	252	179	182	164	121	145	143		200	488	305	331	271
Population B	711	83	259	263	191	186	161	116	132	140		187	200	287	262	241
Population B	711	89	250	257	183	178	164	116	132	143		198	206	284	270	258
Population B	711	112	254	252	179	182	164	121	145	143		200	488	305	329	266
Population B	712	83	259	263	191	186	161	116	132	140		187	200	287	260	243
Population B	712	89	250	257	183	178	164	116	132	143		198	206	284	270	258
Population B	712	112	254	252	179	182	164	121	145	143		200	488	305	329	268
Population B	713	83	259	263	191	186	161	116	132	140		187	200	287	262	243
Population B	713	89	250	257	183	178	164	116	132	143		198	206	284	270	258
Population B	713	112	254	252	179	182	164	121	145	143		200	488	305	329	268
Population B	714	83	259	263	191	186	161	116	132	140		187	200	287	264	243
Population B	714	89	250	257	183	178	164	116	132	143		198	206	284	270	258
Population B	714	112	254	252	179	182	164	121	145	143		200	488	305	329	268
Population B	715	83	259	263	191	186	161	116	132	140		187	200	287	262	243
Population B	715	89	250	257	183	178	164	116	132	143		198	206	284	270	258
Population B	715	112	254	252	179	182	164	121	145	143		200	488	305	329	268
Population B	733	83	259	263	191	186	161	116	132	140		187	200	287	281	245
Population B	733	89	250	257	183	178	164	116	132	143		198	206	284	270	256
Population B	733	112	254	252	179	182	164	121	145	143		200	488	305	329	271
Population B	718	83	259	263	191	186	161	116	132	140		187	202	287	266	233
Population B	718	89	250	257	181	178	164	116	132	147		182	204	284	241	245
Population B	718	112	254	252	179	186	164	121	145	143		192	484	295	351	273
Population B	720	83	259	263	191	186	161	116	132	140		187	202	287	259	223
Population B	720	89	250	257	181	178	164	116	132	147		182	209	284	241	211
Population B	720	112	254	252	179	186	164	121	145	143		192	488	295	345	271
Population B	721	83	259	263	191	186	161	116	132	140		187	202	287	264	233
Population B	721	89	250	257	181	178	164	116	132	147		182	204	284	241	245
Population B	721	112	254	252	179	186	164	121	145	143		190	484	295	351	271
Population B	731a	83	259	263	191	186	161	116	132	140		187	202	287	249	213
Population B	731a	89	250	257	181	178	164	116	132	147		184	204	284	241	260
Population B	731a	112	254	252	179	186	164	121	145	143		192	486	295	366	273
Population B	727	83	259	263	191	186	161	116	132	140		187	202	287	249	213
Population B	727	89	250	257	181	178	164	116	132	147		184	204	284	241	213
Population B	727	112	254	252	179	186	164	121	145	143		192	486	295	368	213
Population B	723	83	259	263	191	186	161	116	132	140		187	192	287	255	223
Population B	723	89	250	257	175	178	164	116	132	143		205	204	290	257	223
Population B	723	83	254	252	179	186	164	123	148	143		205	484	301	362	268
Population B	723a	83	259	263	191	186	161	116	132	140		187	192	287	255	223
Population B	723a	89	250	257	175	178	164	116	132	143		205	204	290	257	223
Population B	723a	83	254	252	179	186	164	123	148	143		205	484	301	362	268
Population B	725	83	259	263	191	186	161	116	132	140		187	192	287	255	223
Population B	725	89	250	257	175	178	164	116	132	143		205	204	290	257	223
Population B	725	83	254	252	179	186	164	123	148	143		205	484	301	362	268
Population B	plus SB	83	259	263	191	186	161	116	132	140		187	192	287	255	223
Population B	plus SB	89	250	257	175	178	164	116	132	143		205	204	290	257	223
Population B	plus SB	83	254	252	179	186	164	123	148	143		205	484	301	362	268
