# Supplementary material for: A Comparison of Entropic Diversity and Variance in the Study of Population Structure
Source: Entropy (Basel). 2023 Mar 13;25(3):492. doi: 10.3390/e25030492 (PMC10048111; doi:10.3390/e25030492)
Supplement: Supplementary file 1 [file entropy-25-00492-s001.zip › entropy-1939759-supplementary/Supplemental Table S1.pdf]

Eric F. Karlin   A comparison of diversity and variance in the studyof genetic structure of populations

Supplemental Table S1. Theoretical maximum and minimum values for allelic diversity and variance.

Each data set (i.e. pair of populations) has the following properties: ten samples per population; no allelic overlap between each of the two populations, equal AMD (Δ) and equal variance within each population, and with 50% of the total (Δ_T) residing in each population.

Minimum

one allele for entire data set (1 marker)
two diploid pops, each with 10 samples

AMDA

Source of Information	Degrees Freedom DF	Diversity Estimate exp(sH)	[0,1] Scaled Diversity D'	Δ	Ω
**AP**	1	1.00	0.00	0.0	0.00
**AI**	18	1.00	0.00	0.0	0.00
**WI**	20	1.00	0.00	1.0	1.00
**Total**	39	1.00	0.00	1.0	1.00

AMOVA

Source	df	SS	MS	Est. Var.	%
**AP**	1	0.00	0.00	0.00	0%
**AI**	18	0.00	0.00	0.00	0%
**WI**	20	0.00	0.00	0.00	0%
**Total**	39	0.00		0.00	0%

one allelel for entire data set (10 markers)
two diploid pops, each with 10 samples

Stat	Value
Ω_{ST}	0.00
Ω_{IS}	0.00
Ω_{IT}	0.00

Stat	Value
F_{ST}	#N/A
F_{IS}	#N/A
F_{IT}	#N/A

Maximum (Δ_{TMAX})

all alleles differ (1 marker)
two diploid pops, each with 10 samples

Source of Information	Degrees Freedom DF	Diversity Estimate exp(sH)	[0,1] Scaled Diversity D'	Δ	Ω	Estimated Probability P(rand >= data)	Source	df	SS	MS	Est. Var.	%
**AP**	1	2.00	1.00	20.0	0.50	1.00	**AP**	1	0.50	0.50	0.00	0%
**AI**	18	10.00	1.00	18.0	0.45	1.00	**AI**	18	9.00	0.50	0.00	0%
**WI**	20	2.00	1.00	2.0	0.05	1.00	**WI**	20	10.00	0.50	0.50	100%
**Total**	39	40.00	1.00	40.0	1.00		**Total**	39	19.50		0.50	100%

Stat	Value	P(rand >= data)	Stat	Value	P(rand >= data)
Ω_{ST}	0.50	1.00	F_{ST}	0.00	1.00
Ω_{IS}	0.90	1.00	F_{IS}	0.00	1.00
Ω_{IT}	0.95	1.00	F_{IT}	0.00	1.00
