# Supplementary material for: A Comparison of Entropic Diversity and Variance in the Study of Population Structure
Source: Entropy (Basel). 2023 Mar 13;25(3):492. doi: 10.3390/e25030492 (PMC10048111; doi:10.3390/e25030492)
Supplement: Supplementary file 1 [file entropy-25-00492-s001.zip › entropy-1939759-supplementary/Supplemental Table S2.pdf]

Eric F. Karlin. A comparison of entropic diversity and variance in the study of population structure

Supplemental Table S2. This table summarizes range of values and expected behavior of the statistics used in this paper. Most of the range values for each index are taken from [27]. Range values for indices not covered by that source are provided by the author and are listed in **bold** font. Behavior is based the results of this study as well as the author's extensive experience gained from other studies. K = the number of populations.

Index	Range	Behavior as D_T or N'_a increases
SIDTA		
Ω_{WI}	**[≈0,1]**	decreases
Ω_{AI}	**[0, ≈1]**	increases
Ω_{AP}	**[0,0.5]**	increases
D'_{AP}	[0,1]	increases
D_{AP}	**[1, K]**	Increases
AMOVA		
F_{WI}	**[≈0,1]**	Increases
F_{AI}	**[<0,1]**	Varies, can be highly negative
F_{STv}	[<0,1]	Varies, can be slightly negative
PH_IPT	[<0,1]	Varies, can be slightly negative
F'_{STv}	[<0,1]	Varies, can be highly negative
Heterozygosity		
F_{STh}	[0,1]*	varies
Jost's D	[0,1]	varies
Adjusted Heterozygosity		
G_{ST}	[<0,1]	varies, can be slightly negative
$G'_{ST}N$	[<0,1]	varies, can be slightly negative
D_{EST}	[<0,1]**	varies, can be highly negative
G''_{ST}	[<0,1]	varies, can be highly negative
$G'_{ST}H$	[<0,1]	varies, can be highly negative

* The range of values for F_{STh} listed in [27] was based on the adjusted version. The range listed here is for the unadjusted version. For one marker, adjusted F_{STh} = adjusted G_{ST}.

**The range of values listed for D_{EST} in [27] was a typo and is here corrected.
